# Alpha-synuclein facilitates to form short unconventional microtubules that have a unique function in the axonal transport

**DOI:** 10.1038/s41598-017-15575-3

**Published:** 2017-11-27

**Authors:** Shiori Toba, Mingyue Jin, Masami Yamada, Kanako Kumamoto, Sakiko Matsumoto, Takuo Yasunaga, Yuko Fukunaga, Atsuo Miyazawa, Sakiko Fujita, Kyoko Itoh, Shinji Fushiki, Hiroaki Kojima, Hideki Wanibuchi, Yoshiyuki Arai, Takeharu Nagai, Shinji Hirotsune

**Affiliations:** 1 Department of Genetic Disease Research, Osaka City University Graduate School of Medicine, Asahi-machi 1-4-3 Abeno, Osaka, 545-8585 Japan; 2 Department of Bioscience and Bioinformatics, Faculty of Computer Science and Systems Engineering, Kyushu Institute of Technology, Kawazu 680-4, Iizuka, Fukuoka, 820-850 Japan; 3 JST-SENTAN, 4-1-8, Honcho, Kawaguchi, Saitama, 332-0012 Japan; 4 JST-CREST, 4-1-8, Honcho, Kawaguchi, Saitama, 332-0012 Japan; 5 Graduate School of Life Science, University of Hyogo, 3-2-1 Kouto, Kamigori-cho, Ako-gun, Hyogo, 678-1297 Japan; 6 RSC-University of Hyogo Leading Program Center, RIKEN SPring-8 Center, 1-1-1 Kouto, Sayo-cho, Sayo-gun, Hyogo, 679-5148 Japan; 7 Graduate School of Materials Science, Nara Institute of Science and Technology, 8916-5, Takayama, Ikoma, Nara, 630-0101 Japan; 8 Department of Pathology and Applied Neurobiology, Kyoto Prefectural University of Medicine Graduate School of Medical Sciences, Kajii-cho, Kawaramachi-Hirokoji, Kamigyo-ku, Kyoto, 602-8566 Japan; 9 Advanced ICT Research Institute, National Institute of Information and Communications Technology, 588-2 Iwaoka, Nishi-ku, Kobe, 651-2492 Japan; 10 Department of Pathology, Osaka City University Graduate School of Medicine, Asahi-machi 1-4-3 Abeno, Osaka, 545-8586 Japan; 11 Department of Biomolecular Science and Engineering, Institute of Scientific and Industrial Research, Osaka University, Mihoga-oka 8-1, Osaka, 567-0047 Japan

## Abstract

Although α-synuclein (αSyn) has been linked to Parkinson’s disease (PD), the mechanisms underlying the causative role in PD remain unclear. We previously proposed a model for a transportable microtubule (tMT), in which dynein is anchored to a short tMT by LIS1 followed by the kinesin-dependent anterograde transport; however the mechanisms that produce tMTs have not been determined. Our *in vitro* investigations of microtubule (MT) dynamics revealed that αSyn facilitates the formation of short MTs and preferentially binds to MTs carrying 14 protofilaments (pfs). Live-cell imaging showed that αSyn co-transported with dynein and mobile βIII-tubulin fragments in the anterograde transport. Furthermore, bi-directional axonal transports are severely affected in αSyn and γSyn depleted dorsal root ganglion neurons. SR-PALM analyses further revealed the fibrous co-localization of αSyn, dynein and βIII-tubulin in axons. More importantly, 14-pfs MTs have been found in rat femoral nerve tissue, and they increased approximately 19 fold the control in quantify upon nerve ligation, indicating the unconventional MTs are mobile. Our findings indicate that αSyn facilitates to form short, mobile tMTs that play an important role in the axonal transport. This unexpected and intriguing discovery related to axonal transport provides new insight on the pathogenesis of PD.

## Introduction

Microtubules (MTs) represent the main cytoskeletal components in eukaryotic cells and consist of globular αβ-tubulin dimers stacked head-to-tail to form protofilaments (pfs) that run lengthwise towards the cell wall, thereby conferring structural polarity^1^. The polymerization dynamics allow MTs to adopt spatial arrangements that can rapidly change in response to cellular needs and perform mechanical works in certain cases^1–3^. *In vitro* polymerized MTs contain various numbers of pfs (10~17 pfs)^4,5^. This heterogeneity is inconsistent with the uniform 13-pfs MTs observed in the majority of cell types^6^. Most MTs with more or less than 13-pfs contain supertwisted pfs that causes motor proteins to wrap helically around the MTs during intracellular transport^4^. The disadvantages of such spiraling may explain the preponderance of 13-pfs MTs in most cells. The MTs are also the main component of macromolecular machineries such as the mitotic spindle, centrioles/basal bodies, cilia and flagella. Importantly, MTs serve as tracks for 2 families of motor proteins, kinesins and dyneins, which move toward the plus and minus ends of MTs, respectively^7–11^, thus facilitating long-range transport within cells. Although numerous kinesins have evolved to manage various transport needs, a single cytoplasmic dynein performs just as many diverse transport activities^12^. To ensure this functional versatility, cytoplasmic dynein is regulated by a number of multifunctional adaptors, including dynactin, BicD, NDEL1, and LIS1^13^. Mice with an inactive *Lis1* allele exhibit cortical, hippocampal and olfactory bulb disorganization caused by delayed neuronal migration, whereas homozygous null mice die early in embryogenesis soon after implantation^14^. Previously, we proposed a model in which LIS1 anchors cytoplasmic dynein on a transportable microtubule (tMT), and together with NDEL1 and mammalian NudC (mNudC), cooperatively regulate the anterograde transport of cytoplasmic dynein in a kinesin-dependent manner^15,16^.

A previous series of experiments suggested the presence of MTs specialized for the anterograde transport of cytoplasmic dynein. An earlier report suggested that cytoplasmic dynein mainly associates with the slow component b complex during anterograde transport^17^, at a speed of 2–8 mm/day^18^, suggesting a requirement of 3–12 months for the anterograde transport of cytoplasmic dynein through a human femoral nerve. In contrast, our previous work suggested that the anterograde transport of cytoplasmic dynein depends on fast transport mediated by kinesin-1^15^. In addition, we demonstrated that when moving in the anterograde direction, cytoplasmic dynein binds MTs that are stable and relatively tiny fragments, which is consistent with pioneering work in this area^19^. Compared with cytoskeletal MTs, the components and structures of fast-moving tiny MT fragments are not well understood. The general instability of these tiny MTs suggests that special mechanisms, such as posttranslational modification, are required for their stabilization. MT dynamic organization and stability are also heavily modulated by MT-associated proteins (MAPs)^20^, such as MAP1a, MAP1b, MAP2a-c, MAP4, and tau protein. Therefore, we wished to explore and identify the MAPs essential for the creation of the short tMTs that are required for the anterograde transport of cytoplasmic dynein.

In this study, our proteomic analyses identified α-synuclein (αSyn) and γ-synuclein (γSyn) as transported components from rat femoral nerve extraction. Synucleins (Syns) were originally cloned from purified cholinergic vesicles of the *Torpedo* electric organ, in which they are localized within the cell nuclei and presynaptic nerve terminals^21^. Because of the importance in autosomal dominant Parkinson’s disease (PD)^22^, the physiological functions of αSyn and the mechanism underlying neuronal degeneration have been intensively investigated. Recently, αSyn was found to play an important role in the maintenance of synaptic vesicles in presynaptic terminals^23,24^. In addition, αSyn has been shown to be functionally relevant for MTs, since the polymerization of purified tubulin into MTs is lost in the presence of its mutations^25^. αSyn is also known to be involved in the endocytosis and exocytosis of synaptic vesicles via MT assembly^26^, although the causative mechanism of PD remains unclear.

Here, we found that αSyn plays a unique function in the anterograde transport of cytoplasmic dynein. Notably, siRNA-mediated depletion of Syns significantly impaired the bi-directional axonal transports. In addition, our study provides evidence for the occurrence of tMTs, a novel type of MT fragments with structural features that differ from those of regular MTs and are specialized for intracellular transport. Aligning our findings with those of previous pathological and genetic studies, we propose a novel etiological mechanism in PD that is attributable to biased αSyn distribution and defective intracellular transport due to the loss of normal interactions between mutated αSyns and tMTs.

## Results

### Identification of synuclein proteins as transported components

**Figure 1:**
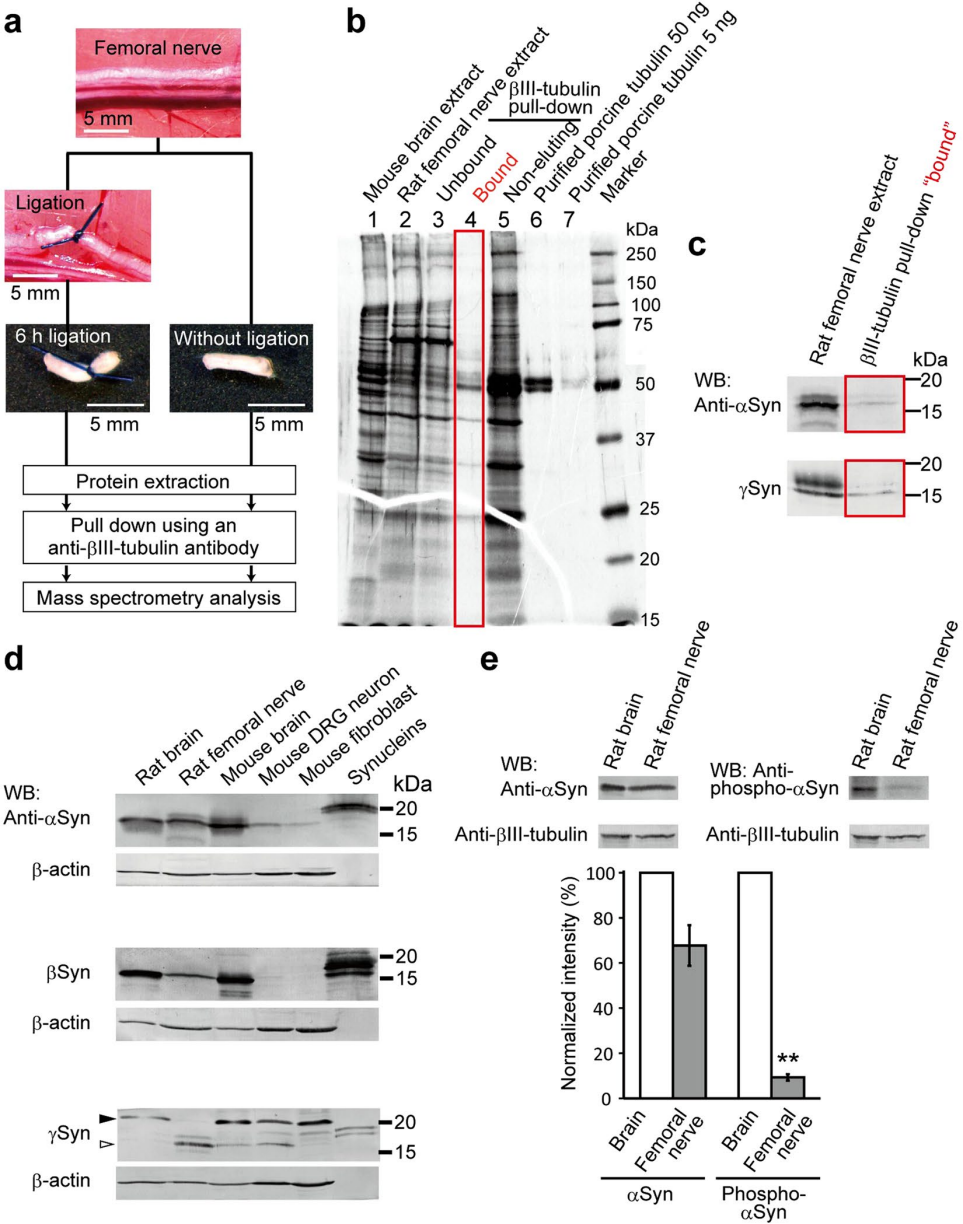
αSyn and γSyn interact with βIII-tubulin in rat femoral nerves. (**a**) Schematic illustration of protein extraction from the rat femoral nerve. Under anesthesia, both sides of the rat femoral nerve roots were ligated to promote the accumulation of transported components at the ligated terminal. After 6 h of ligation, soluble proteins were extracted and subjected to a pull-down assay using an anti-βIII-tubulin antibody. After elution, the components were analyzed by LC-MS/MS. Scale bar: 5 mm. (**b**) Overview of silver staining with femoral nerve extraction. The eluted co-precipitates are indicated by the red square (lane 4). (**c**) Examination of co-precipitates by Western blotting (WB). According to the βIII-tubulin interactome obtained by LC-MS/MS analysis (Supplementary Fig. S1a), the co-precipitates were examined with anti-αSyn (upper) and anti-γSyn (lower) antibodies. (**d**) Expression profile of the three synucleins (Syns) examined in rat and mouse as indicated at the top. The filled arrowhead in the γSyn detection panel indicates phosphorylated γSyn, and the open arrowhead indicates unphosphorylated γSyn. Recombinant αSyn, βSyn and γSyn were loaded in the right-hand lane in each panel. β-actin was used as a loading control. (**e**) Phosphorylated αSyn in rat brain and femoral nerve. The expression of αSyn and phosphorylated αSyn was probed with anti-αSyn and anti-phospho-S129-αSyn antibodies, respectively. βIII-tubulin was used as a loading control. Quantification of the phosphorylation observed in 3 independent sets of experiments is shown in the lower panel. The signal intensity of αSyn or phospho-αSyn in brain was set at 100%. Data are presented as the mean ± SEM. **P < 0.01 by Student’s *t*-test. See also Supplementary Fig. S1.

We previously proposed a model in wich LIS1 anchors cytoplasmic dynein on a short transportable microtubule (tMT), which is followed by the anterograde transport by kinesin-1 under mammalian NudC (mNudC) mediation^15,17^.To confirm this speculation and identify strong candidates for the actors in the model, we ligated rat femoral nerves to interrupt and accumulate axonally transported components. The ligated femoral nerves were then subjected to proteomic analysis (Fig. 1a). The femoral nerve is a large nerve in which axons form large bundles with the same polarity. For this reason, microtubules (MTs) in an axon have uniform orientations in which their plus ends face the neurite tip and minus ends face the soma^27^. Accordingly, kinesin motors accumulate at the proximal side of the ligated femoral nerve, whereas cytoplasmic dynein motors accumulate in both proximal and distal to a ligation. It is reasonable to assume that tMTs would accumulate with kinesin on the proximal side of the ligation point. To explore this, extracted lysates from the ligated fragments containing transported components were subjected to a pull-down assay using an antibody against βIII-tubulin, a neuron-specific isoform (Fig. 1b). The co-precipitated components were analyzed by liquid chromatography-tandem mass spectrometry (LC-MS/MS), and α-synuclein (αSyn) and γ-synuclein (γSyn) were detected as transported components (Supplementary Fig. S1a). Western blotting (WB) analysis also confirmed the presence of αSyn and γSyn in the ligated fragments (Fig. 1c).

To address the function of αSyn as a regulator of MT dynamics, we first characterized the expression patterns of synucleins (Syns) in neurons and in mouse embryonic fibroblasts (MEFs). Interestingly, αSyn and β-synuclein (βSyn) displayed a biased distributions and were more strongly expressed in neurons than in non-neuronal cells, whereas γSyn was detected in all examined cell types with two different protein sizes (Fig. 1d and Supplementary Fig. S1b). γSyn belongs to a novel class of G protein-coupled receptor kinase (GRK) substrates that can be recognized by their substantially decreased electrophoretic mobility^28^. Indeed, γSyn was found to be predominantly phosphorylated in both brain tissue and MEF cells (Fig. 1d, filled arrowhead). Curiously, γSyn was exclusively unphosphorylated in femoral nerves (Fig. 1d, open arrowhead). To confirm γSyn phosphorylation, we treated brain extracts with alkaline phosphatase and observed a shift in the electrophoretic mobility of γSyn (Supplementary Fig. S1c). We also examined the phosphorylation of αSyn in the brain and in femoral nerves, as the majority of αSyn within Lewy bodies of patients with PD and related synucleinopathies is phosphorylated at serine residue 129 (pS129)^29^. As shown in Fig. 1e, the phosphorylated αSyn detected by an anti-pS129 antibody does not display altered electrophoretic mobility. The distribution of phosphorylated αSyn in femoral nerves was significantly reduced compared with that of the non-phosphorylated form (Fig. 1e). These data suggest that regulation of the phosphorylation of αSyn and γSyn seems to be important for their function in nerve axons.

We next examined the interactions of tubulin with Syns via a Biacore analysis. Immobilized tubulin on the sensor chip was incubated with each recombinant Syn, and the measured K_d_s were 3.90 × 10^−6^ for αSyn, 1.16 × 10^−5^ for βSyn, and 2.97 × 10^−5^ for γSyn, indicating that wild type (WT) Syns interact with tubulin dimers directly (Supplementary Fig. S2a–d,i). We next examined the effects of the αSyn mutations A30P^30^ and E46K^31^, which are known to cause inherited forms of PD. These mutations completely suppressed the binding affinity of αSyn to tubulin (Supplementary Fig. S2e–f,i). To explore the effect of phosphorylated αSyn at S129, we examined the affinity of αSyn S129E and S129A for tubulin and found that S129E but not S129A also eliminated the binding potential of αSyn to tubulin (Supplementary Fig. S2g–i).

αSyn cellular inclusions consist of fibrils of polymerized αSyn. Furthermore, *in vitro*, αSyn intrinsically polymerizes into fibrils that resemble the authentic filaments present in pathological lesions^32,33^. To address the interaction potential of Syns, we examined their binding affinity for each other, and no appreciable binding affinities between Syns were detected under our conditions (Supplementary Fig. S2i–l). Thus each Syn protein individually binds to tubulin, as described in previous reports^25,34^. Inconsistent with a previous report^35^, we successfully detected interactions between WT Syns and polymerized MTs using a MT pelleting assay (Supplementary Fig. S2m). In this assay, αSyn mutants except for S129A did not show binding affinity to MTs (Supplementary Fig. S2n), which is consistent with the results from the Biacore analyses (Supplementary Fig. S2).

### Dynamic transport of Syns in DRG neurons

**Figure 2:**
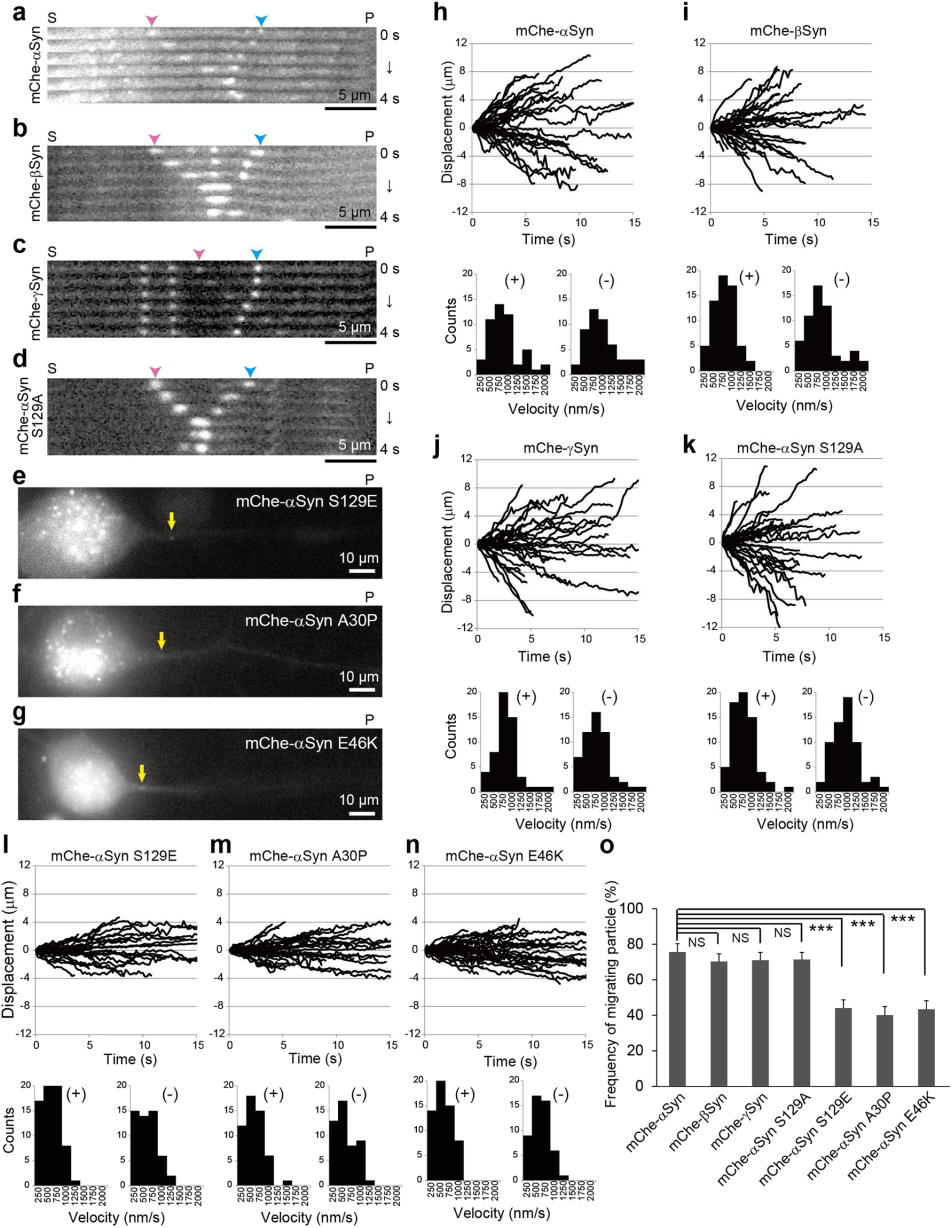
Transport behavior of mCherry-tagged Syns in DRG neurons. (**a**–**d**) mCherry-tagged Syns were expressed in DRG neurons using lentivirus vectors and examined using live-cell imaging. The kymographs depict moving particles of mCherry-αSyn (**a**), mCherry-βSyn (**b**), mCherry-γSyn (**c**), and mCherry-αSyn S129A (**d**) along axons. Pink and blue arrowheads indicate anterograde and retrograde movements, respectively. The elapsed time is shown at the right. (**e**–**g**) Abnormal accumulation of three mutated αSyns in the DRG soma. Lentivirus vector-mediated expression of mCherry-αSyn S129E (**e**), mCherry-αSyn A30P (**f**), and mCherry-αSyn E46K (**g**) was observed, and all three mutated αSyns were found to aberrantly accumulate in the soma. The yellow arrows indicate punctate intensity in the images showing the disrupted movement of the αSyn mutated forms. In (**a**–**g**), ‘S’ and ‘P’ indicate the directions of the neuronal soma and its peripheral region, respectively. Scale bars: 5 μm in (**a**–**d**) and 10 μm in (**e**–**g**). (**h**–**n**) Analyzed trajectories of mCherry-tagged Syns as indicated at the upper areas of the panels. Anterograde and retrograde displacements are shown as positive and negative values, respectively (N = 30 particles in each graph). The distribution of velocities is shown beneath each trajectory (N = 60 particles). “ + ” and “−” in each graph indicate anterograde and retrograde movement, respectively. (**o**) Frequencies of moving particles as indicated on the transverse axis (N = 60 in each target). Particles with velocities > 500 nm/s were defined as moving. *P* values were calculated with *t*-test. ***p < 0.001, mean ± SEM; NS indicates not significant. See also Supplementary Videos 1–2.

MTs are decorated by MAPs *in vivo*; consequently, MT assembly is controlled in specific ways depending on organism development, stage specification, shape determination, and cell division^20^. Although MTs are dynamic structures, MAP-decorated MTs exhibit short-term stability. Accordingly, MAPs are generally considered as immobile. However, a previous report indicated that αSyn is dynamically transported in hippocampal neurons^36^. To determine the mobility of Syns, we monitored the dynamics of Syn proteins in DRG neurons. We expressed enhanced monomeric red fluorescent protein, mCherry (mChe)-tagged Syn in DRG neurons and traced its movement using a live-cell imaging system. Exogenously tagged Syns exhibited distributions similar to those of endogenous Syns (Supplementary Fig. S3a–f), indicating the suitability of mChe-tagged Syns for tracking the behaviors of endogenous Syns. Live-cell imaging revealed that αSyn, βSyn, and γSyn were highly and bi-directionally mobile in the neurites of DRG neurons (Fig. 2a–c,h–j,o and Supplementary Video 1). The mean velocity of αSyn, βSyn, and γSyn particles during their bidirectional movements in the neurite was approximately 0.7 μm/s (Fig. 2h–j), which is similar to the normal rate of movement of kinesin-1 and cytoplasmic dynein^10,11^. However, αSyn mutations except for S129A exhibited significantly decreased rate of transport in both directions, and displayed abnormal accumulations within the soma (Fig. 2d–g,k–o, and Supplementary Video 2).

We further investigated the behaviors of other MAPs, including tau and doublecortin (DCX). Tau protein is a highly soluble MAP that modulates the stability of axonal MTs, and the mutation of this protein is associated with a form of frontotemporal dementia^37^. On the other hand, DCX was discovered in a screen of patients with subcortical band heterotopia and X-linked lissencephaly^38,39^. In the neurites, mChe-tau displayed a punctate distribution on a fibrous background, which is similar to the distribution of endogenous tau (Supplementary Fig. S3g–i). Curiously, only the punctate mChe-tau was highly mobile (Supplementary Video 3) as described in a previous report^40^. However, mChe-DCX exhibited only a fibrous expression and did not show any punctate distributions or moving particles (Supplementary Fig. S3j–l and Supplementary Video 3). Tau binds MTs carrying 14-pfs as well as 13-pfs^41^, whereas DCX prefers MTs that have 13-pfs^42,43^. Based on these findings, it is reasonable to hypothesize that moving particulate Syns might selectively bind to mobile MTs, and this mobile MTs may contain an unconventional number of 14-pfs.

### Co-migration of synuclein particles with cytoplasmic dynein during anterograde movements

**Figure 3:**
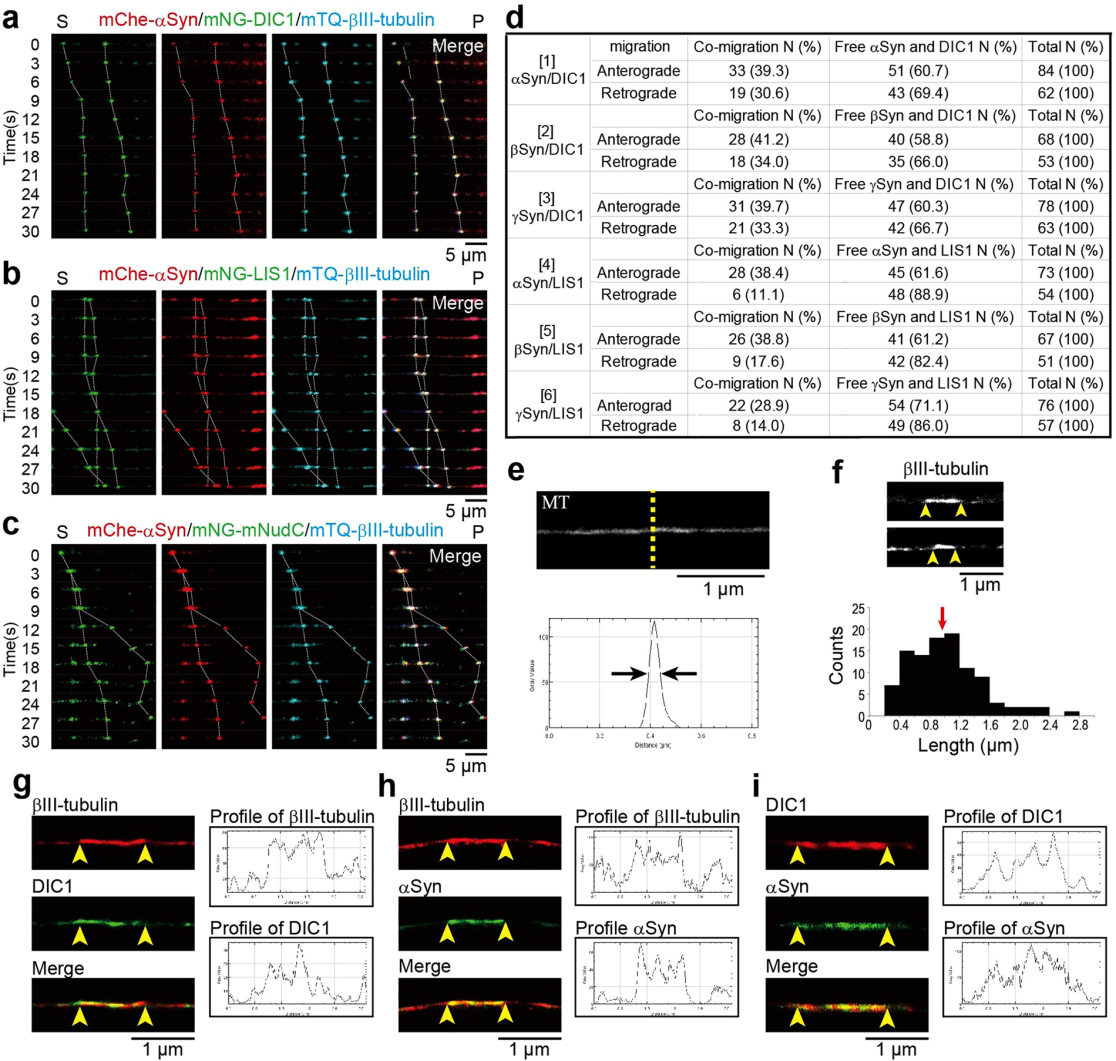
Triple- and dual-color imaging showing the co-migration of αSyn with mobile MTs. (**a**–**c**) Direct visualization of the anterograde movement of fluorescently tagged proteins in DRG neurons using triple-color live-cell imaging. The co-migration of mNG-DIC1, mChe-αSyn and mTQ-βIII-tubulin (**a**), mNG-LIS1, mChe-αSyn and mTQ-βIII-tubulin (**b**), and mNG-mNudC, mChe-αSyn and mTQ-βIII-tubulin (**c**) was observed using confocal time-lapse microscopy. The dotted lines indicate dynamic co-migration of the three types of particles as indicated in each image set. ‘S’ and ‘P’ indicate the directions of the neuronal soma and its peripheral region, respectively. Scale bar: 5 μm. (**d**) Anterograde and retrograde co-migration frequencies in DRG neurons. Co-migration (left), independent migration (middle), and total number of examined signals (right) are indicated. (**e**) Super-resolution photoactivated localization microscopy (SR-PALM) image of MTs. The intensity distribution profile across a MT at the area indicated by the yellow dotted line is shown in the graph. The width at the half-value of the peak approaches ~50 nm (indicated by the black arrows). (**f**) Short MT fragments detected along axons. The ends of the fragments are indicated by yellow arrowheads. The measured length of the MT fragments are shown beneath the image. The median length is 1 μm (red arrow). (**g**–**i**) Co-localization of DIC1 and αSyn with βIII-tubulin detected by dual-color SR-PALM. Co-localization of cage 590-labeled βIII-tubulin with cage 500-labeled DIC1 (**g**), cage 590-labeled βIII-tubulin with cage 500-labeled αSyn (**h**), and cage 590-labeled DIC1 with cage 500-labeled αSyn (**i**) were examined. The intensity profiles are shown at the right side of each panel. Scale bar: 1 μm in (**e**–**i**). See also Supplementary Fig. S4 and Supplementary Video 4.

In our model, LIS1 anchors dynein to a tMT to form a LIS1–dynein–tMT complex that is transported by kinesin-1 to the plus ends of MTs via mNudC mediation^15,16^. To determine whether the tMTs involved in anterograde cytoplasmic dynein transportation correspond to Syn-bound mobile MT fragments, we examined the dynamic co-migration of dynein, LIS1 and Syns using triple-color live-cell imaging. We first examined the co-migration of mChe-αSyn, mNG-DIC1 and mTQ-βIII-tubulin (Fig. 3a and Supplementary Video 4) and found that all 3 of these proteins clearly co-migrated during anterograde movement. We subsequently investigated the co-migration of mChe-αSyn, mNG-LIS1 and mTQ-βIII-tubulin. Again, all 3 proteins co-migrated during anterograde movement (Fig. 3b and Supplementary Video 4). Expectedly, the co-migration of mChe-αSyn, mNG-mNudC and mTQ-βIII-tubulin was also observed (Fig. 3c and Supplementary Video 4), which is consistent with our previous report concerning the function of mNudC as an adaptor protein for kinesin transport^16^. Taken together, our data indicate that αSyn, dynein, LIS1, mNudC and βIII-tubulin co-migrate anterogradely with kinesin. We further measured the co-migration frequency of each of the three Syns with DIC and LIS1 (Fig. 3d), and found that these proteins co-migrated during both retrograde and anterograde movement but that their co-migration occurred at a lower frequency during retrograde movement. The reduction in frequency of retrograde co-migration was most pronounced for Syns and LIS1 (Fig. 3d). We previously demonstrated that more than 50% of LIS1 is degraded at the cell cortex via calpain-dependent proteolysis after transport to the MT plus ends^44^. Presumably, the observed reduction in the retrograde co-migration frequency of Syns and LIS1 can be attributed to the calpain-dependent proteolysis of the LIS1 protein.

αSyn, a presynaptic protein, associates with the distal reserve pool of synaptic vesicles^45^. To investigate whether the co-migrating complexes observed by triple-color live-cell imaging (Fig. 3a–d) are mediated by tMTs or membrane-bound organelles, we characterized the structural features of co-transporting units using super-resolution structured illumination microscopy (SR-SIM) in combination with super-resolution photo-activated localization microscopy (SR-PALM). SR-SIM images showed αSyn particles, many of which co-localized with mTQ-βIII-tubulin labelled MTs, as well as DIC1, LIS1 and mNudC (Supplementary Fig. S4). The ultrastructure of the endogenous of βIII-tubulin, αSyn and cytoplasmic dynein was also investigated by SR-PALM. The cross-sectional profile of MTs obtained by SR-PALM achieved a 50 nm resolution, which is sufficient to permit the discrimination of MT fragments from membrane-bound organelles (Fig. 3e). Indeed, the SR-PALM analysis clearly showed that filamentous βIII-tubulin fragments are associated with cytoskeletal MTs with 0.2–2.4 μm in length (Fig. 3f). The *in vitro* flexural rigidity of MTs corresponds to a persistence length ranging from 20 μm to 10 mm^46,47^, which shows that a MT is a rigid polymer, and indicates that the partially increased signals of βIII-tubulin obtained by SR-PALM are attributable to the true overlapping of two types of MTs rather than by simple bending (Fig. 3f). Furthermore, dual colored SR-PALM displayed co-localization of endogenous βIII-tubulin with αSyn and DIC1 in a fibrous fashion, indicating that these components form a direct complex rather than undergoing a membrane mediated indirect interaction (Fig. 3g–i and Supplementary Fig. S5). In addition, the effect of detergent treatment on the interaction of βIII-tubulin and αSyn was also examined using a pull-down assay, and no effect was detected under our experimental conditions (Supplementary Fig. S6). These findings show that the co-migrating complexes are tMT fragments rather than membrane-bound organelles.

### Effects of αSyn and γSyn on axonal transport

**Figure 4:**
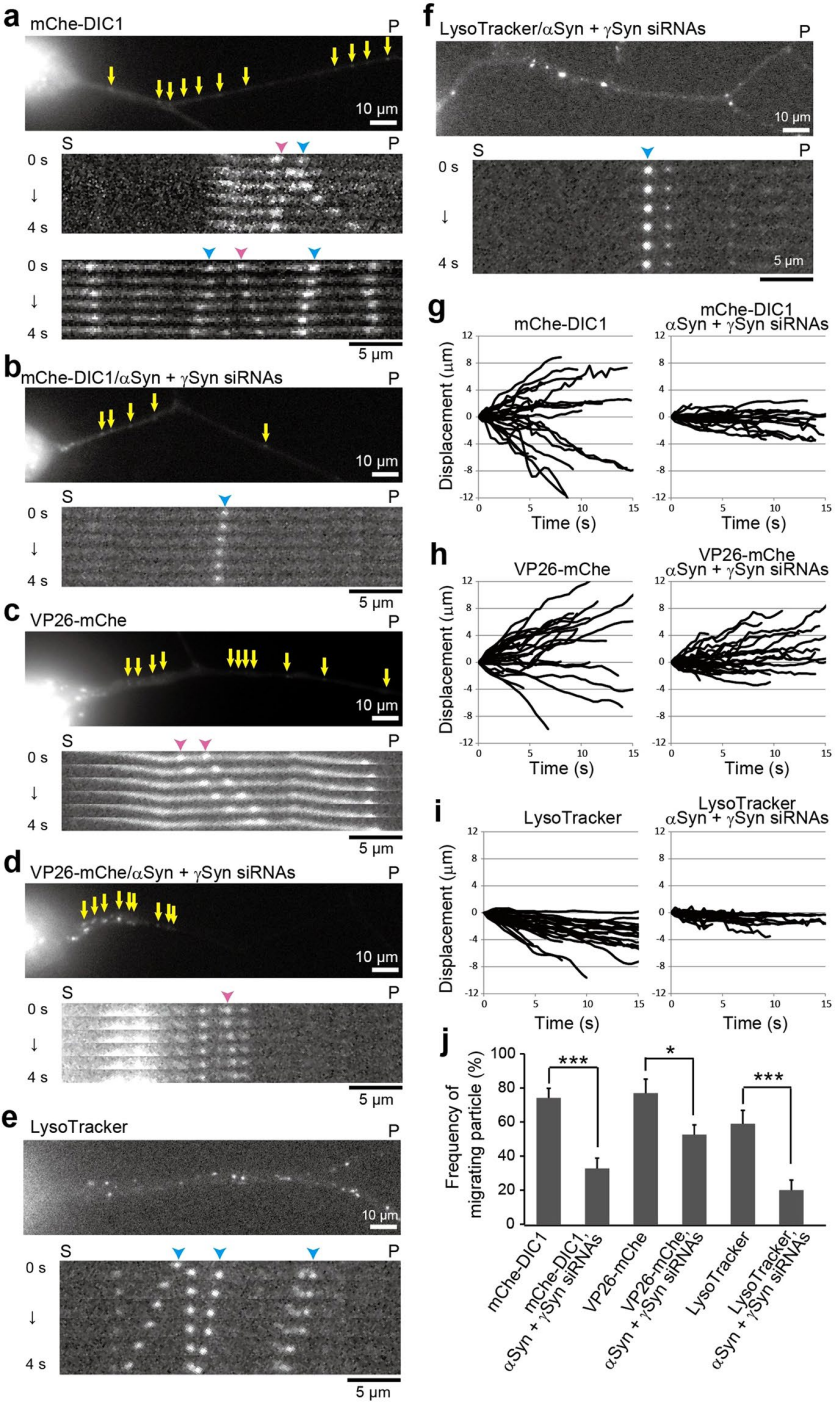
Effect of αSyn and γSyn on axonal transport. (**a**–**f**) Axonal transport visualized using mCherry-DIC1, VP26-mCherry and LysoTracker. The behavior of mCherry-DIC1 (**a**,**b**), VP26-mCherry (**c**,**d**) and LysoTracker (**e**,**f**) in the axon was observed with (**b**,**d** and **f**) or without (**a**,**c** and **e**) treatment of αSyn and γSyn siRNAs. Representative kymographs are shown beneath each image, and the elapsed time is shown on the left. mCherry-DIC1 was expressed using the lentivirus infection system, and VP26-mCherry and all siRNAs were introduced using the Neon transfection system. The pink and blue arrowheads indicate dynamic anterograde and retrograde movement, respectively. “S”, soma; “P”, peripheral region. Scale bars: 10 μm in DRG images and 5 μm in kymographs. (**g**–**i**) Summarized trajectories of target particles as indicated. Thirty particles were traced in each case. Positive values indicate anterograde displacement, and negative values indicate retrograde displacement. (**j**) Statistically quantified frequencies of moving particles. Particles moving at > 500 nm/s were quantified (N = 60 for each target). *P* values were calculated with *t*-test, mean ± SEM; *p < 0.05, ***p < 0.001. See also Supplementary Video 5.

Disruption of *Lis1* led to the abnormal accumulation of cytoplasmic dynein in MEF cells^15^, suggesting that the simple diffusion of cytoplasmic dynein is not sufficient for its proper anterograde transport. Therefore, an active anterograde transport system should be present. To determine whether Syns contribute to this process, we performed siRNA mediated depletion of αSyn and γSyn but not of βSyn because of its extremely low expression (Fig. 1d and Supplementary Fig. S7a). We depleted αSyn and γSyn using siRNAs, and examined the effects on the bidirectional axonal transports in DRG neurons. In the axons, mChe-DIC1 exhibited bi-directional movements (Fig. 4a,g,j and Supplementary Video 5). The acute depletion of αSyn and γSyn by targeting siRNAs remarkably reduced the number of mobile mChe-DIC1 particles, leaving only an immobile fraction mChe-DIC1 in DRG neurons (Fig. 4b,g,j and Supplementary Video 5). We previously reported that interaction between cytoplasmic dynein and MTs is required for its anterograde transport^15^. Accordingly, defective anterograde transport of cytoplasmic dynein caused by the depletion of αSyn and γSyn is a primary event that is likely attributable to impaired tMT generation and/or trafficking. Defective retrograde transport is likely a secondary event most probably caused by a deficient supply of cytoplasmic dynein to the plus ends of MTs. The effect of αSyn and γSyn on the anterograde transport was also examined by using VP26, a capsid protein of herpesvirus that is often used for analyzing anterograde transport with live-cell imaging^48^. In DRG neurons, VP26-mChe exhibited bidirectional movements, although its movement was biased in the anterograde direction (Fig. 4c,h,j and Supplementary Video 5). After acute αSyn and γSyn depletion, anterograde movement of VP26-mChe was mildly affected, whereas retrogradely moving particles almost completely disappeared (Fig. 4d,h,j and Supplementary Video 5). For the analysis of retrograde transport, LysoTracker was used as an indicator. Lysosomes predominantly underwent retrograde movement in the axon (Fig. 4e,i,j and Supplementary Video 5). As expected, retrograde movement of lysosomes was severely affected after acute αSyn and γSyn depletion (Fig. 4f,i,j and Supplementary Video 5). Furthermore, knock down of αSyn and γSyn caused the depletion of cytoplasmic dynein at the periphery of the cell (Supplementary Fig. S7b). Our findings support that Syn-bound tMTs have the specialized functions that are essential for the anterograde transport of cytoplasmic dynein and consequently regulate dynein-dependent retrograde transport.

### αSyn regulates the polymerization and depolymerization of MTs

**Figure 5:**
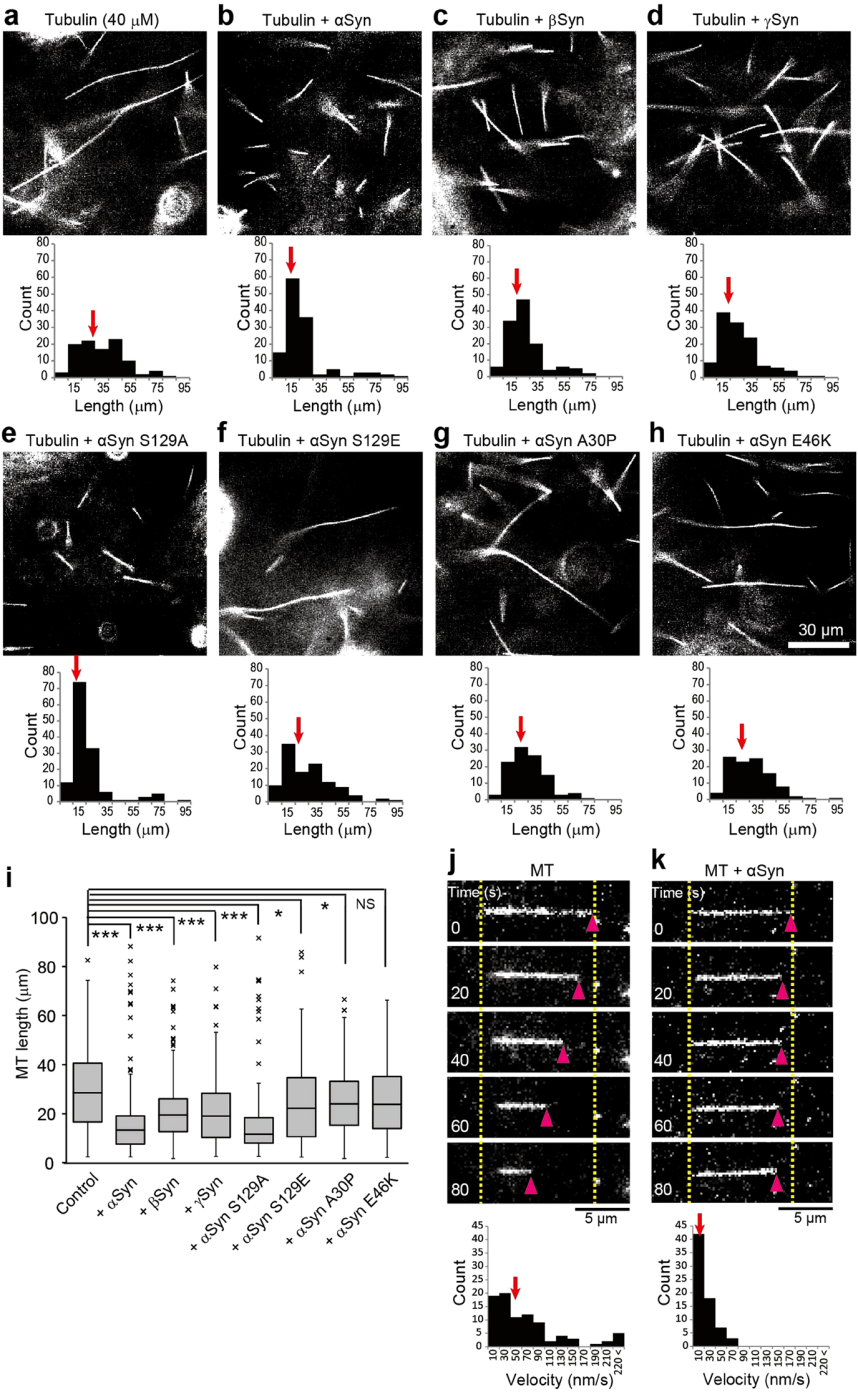
Effect of Syns on tubulin polymerization and depolymerization. (**a**–**h**) MTs undergoing polymerization with unlabeled tubulin *in vitro* were visualized using dark-field light microscopy. Tubulin polymerization was performed without Syns (**a**) and with αSyn (**b**), βSyn (**c**), γSyn (**d**), αSyn S129A (**e**), αSyn S129E (**f**), αSyn A30P (**g**), or αSyn E46K (**h**). MT length with or without Syns was measured and is shown beneath each image. The median values are indicated by red arrows. Scale bar: 30 μm. (**i**) Box-and-whisker plots of the lengths of MTs polymerized *in vitro* (N = 100 for each condition). (**j**–**o**) Effect of αSyns on MT stabilization. Unilaterally occurring spontaneous depolymerization was measured *in vitro* without αSyns (**j**) and with αSyn (**k**), αSyn S129A (**l**), αSyn S129E (**m**), αSyn A30P (**n**), or αSyn E46K (**o**). The distributions of the depolymerization velocities are shown beneath each image set. Spontaneous depolymerization proceeded unilaterally in each case. The pink arrowheads indicate the tips of depolymerizing MTs, and the dotted yellow lines indicate the original lengths of the MTs. The median values are indicated by red arrows. Scale bar: 5 μm. (**p**) Box-and-whisker plots of the spontaneous depolymerization velocities (N = 80 in each condition). *P* values in (**i**) and (**p**) were calculated with *t*-test of nonparametric test, mean ± SEM; ***p < 0.001, **p < 0.01, *p < 0.05, “NS” means not significant. See also Supplementary Videos 6 and 7.

To examine the effect of αSyn on MT dynamics, we performed *in vitro* MT polymerization and depolymerization assays. Fluorescence dye-conjugated tubulin can be used in *in vitro* to visualize MTs; however, the fluorescence moiety of labeled tubulin perturbs the physiological polymerization of tubulin molecules. To avoid this disturbance, we used dark-field light microscopy with unlabeled tubulin at a concentration of 40 µM to examine the self-assembly process of MTs. In the absence of recombinant Syns, the lengths of the assembled MTs varied widely from 5 µm to 95 µm (Fig. 5a,i and Supplementary Fig. S8a). In contrast, the majority of MTs polymerized in the presence of WT Syns were shorter (Fig. 5b–d,i and Supplementary Fig. S8b–d). The effects of αSyn mutations on MT polymerization were also examined. αSyn S129A exhibited an effect similar to that of WT αSyn (Fig. 5e,i and Supplementary Fig. S8e), namely, the generation of numerous short MTs. In clear contrast, αSyn S129E, A30P and E46K had no obvious effects on MT polymerization (Fig. 5f–i and Supplementary Fig. S8f–h). MT nucleation persists throughout most of the polymerization phase, despite the progressive decrease in the free GTP-tubulin concentration^49^. Accordingly, numerous short MTs are formed when tubulin is assembled at higher concentrations. Our data showed that Syns had similar effects on the MT polymerization and that these effects were eliminated by the introduction of αSyn mutations except for the S129A mutation.

To determine whether αSyn suppresses MT disassembly, we designed an improved assay in which biotin-conjugated MTs are anchored at a single point to a coverslip and exhibit pivoting movements, presumably as the result of thermal forces^50^. Each anchored MT was rotated erratically around its anchored point on the glass surface, and this rotation was accompanied by spontaneous depolymerizations (Fig. 5j and Supplementary Video 6). Unexpectedly, this spontaneous depolymerization was significantly inhibited by the addition of αSyn or the αSyn S129A mutant protein, but not by the addition of the S129E, A30P or E46K mutant proteins (Fig. 5j–p, and Supplementary Video 7). The *de novo* assembly of pure tubulin begins after an initial lag phase, after which MTs form rapidly until a plateau level of polymerization is reached. The lag phase occurs because subunits can be much more easily added to an existing MT (elongation) than induced to start a MT *de novo* (nucleation). Presumably, Syn stabilizes the initial MT fragments; therefore, at limited tubulin concentrations, numerous short MTs can form and remain stable.

### Characterization of synuclein-bound MTs by electron microscopy

**Figure 6:**
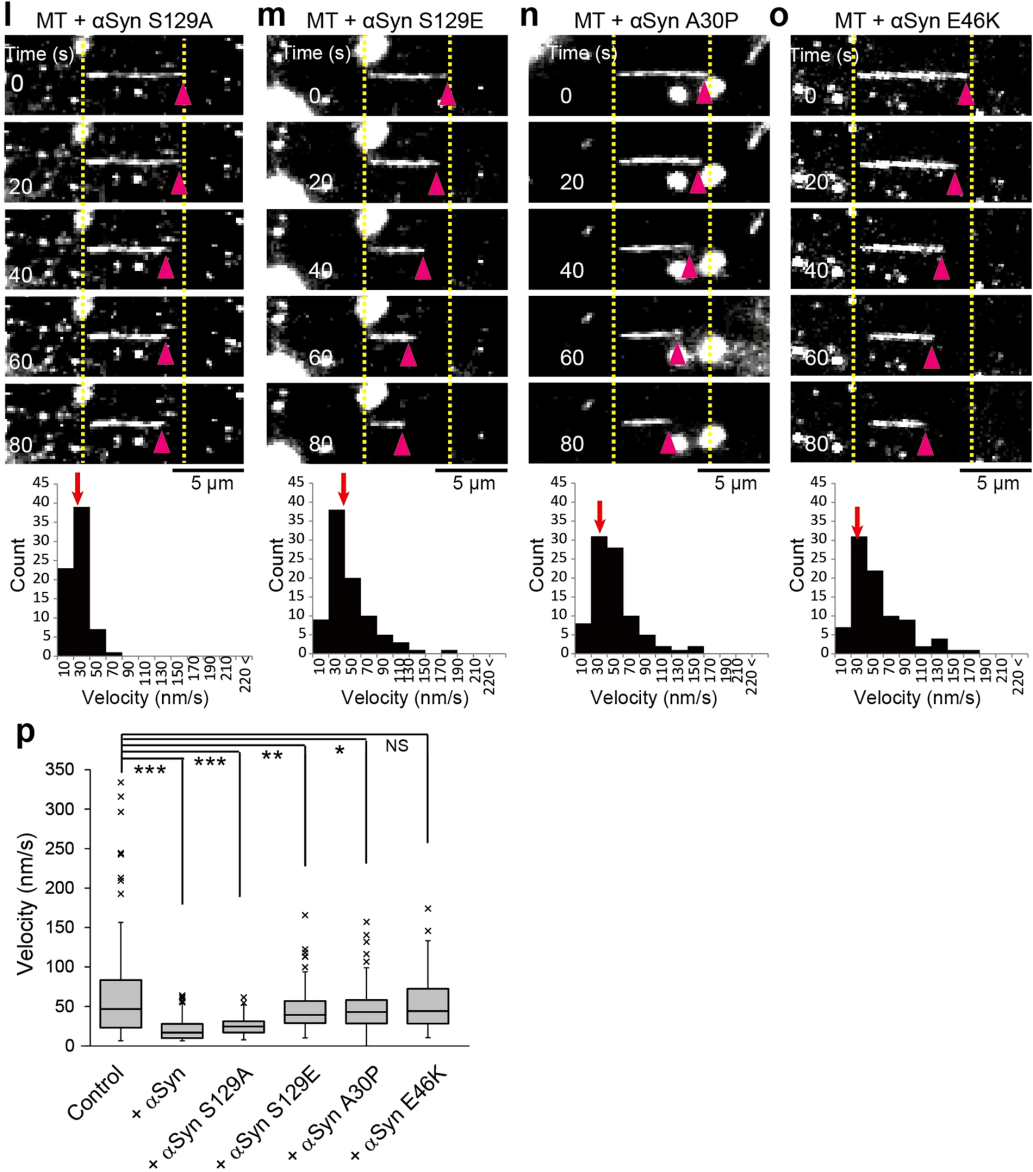
Characterization of αSyn binding to MTs using colloidal gold particles and Halo-tags. (**a**,**b**) αSyn binding to MTs was analyzed by transmission electron microscope (TEM). MTs were mixed with colloidal gold-labeled αSyn (Gold-αSyn) and negatively stained with 2% of uranyl acetate. Gold-αSyn was prepared from N-terminal His-tagged αSyn (His-αSyn). Gold-αSyns appeared as string-like αSyn polymers on MTs. (**c**) TEM image of MT polymerized with Halo-tagged αSyn (Halo-αSyn). Bamboo joint-like structures (indicated by magenta arrowheads) are visible on the MTs. (**d**) MT end structure with Halo-αSyn. Joint-like structures similar to those shown in (**c**) are indicated by magenta arrowheads. Halo-αSyns were also observed in the zone between the outwardly opened tubulin sheet and MT cylinder (blue arrowheads). (**e**) Gold-αSyn located at the transition zone (blue arrowheads). (**f**) MT pull-down assay. mNudC co-precipitated with MTs was examined in the absence and presence of αSyn. (**g**) Dual labeling immunoelectron microscopy (IEM) used to visualize the interaction of MT with mNudC and αSyn. mNudC was labeled with 10 nm colloidal gold (green) via anti-mNudC antibody; and His-αSyn was labeled with 5 nm colloidal gold (red). Co-localization of mNudC and αSyn on a MT is indicated by arrowheads. (**h**) MT polymerized with Halo-αSyn (magenta) and Gold-mNudC (green). Bamboo joint-like structures (magenta) and colloidal gold (green) indicate co-localization of mNudC with Halo-αSyn on a MT. (**i**) Cryo-TEM image of MTs polymerized with Halo-αSyn. Joint-like structures on MTs are indicated by magenta arrowheads. MT pfs numbers determined from Moiré patterns are indicated at the top right. (**j**) Distribution of the pfs numbers of polymerized MTs. The MTs assembled from 40 μM of tubulin (tu) without paclitaxel stabilization mainly formed 13- and 14-pfs MTs (for tu 40 μM, N = 261). The addition of αSyn clearly increased the number of MTs carrying 14-pfs even at 5 μM tubulin (tu 40 μM + αSyn, N = 259; tu 40 μM + Halo-αSyn, N = 135; tu 5 μM + αSyn, N = 111). (**k**) Cryo-TEM image of MTs polymerized with αSyn and 5 μM of tubulin. MT pfs numbers determined from Moiré patterns are indicated at the top right. Bar: 30 nm. (**l**) Selective binding of αSyn to MTs. A mixture of axoneme-nucleated MTs (axoneme-MTs) and GMPCPP polymerized MTs (GMPCPP-MTs) was incubated with TMR-Halo-αSyn (red) in a chamber. The white arrows indicate axoneme-MTs; narrow MTs correspond to GMPCPP-MTs. TMR-Halo-αSyn appears to preferentially bind to GMPCPP-MTs, but not to axoneme-MTs. Scale bar: 30 nm in (**a**–**e**), (**g**–**i**) and (**k**); and 5 μm in (**l**).

We next characterized the structural features of Syn-bound MTs using transmission electron microscopy (TEM). Robust MTs formed *in vitro* at 40 µM of tubulin, whereas polymerization did not occur below the minimum tubulin concentration of 10 µM that is required for its assembly^51^, even when BSA or GST was added (Supplementary Fig. S9a–e). However, the addition of αSyn, βSyn, γSyn or αSyn S129A to 5 µM tubulin solution remarkably facilitated MT assembly, which was not observed with the addition of αSyn S129E, A30P or E46K (Supplementary Fig. S9f–n). To determine the mechanism by which Syns facilitate tubulin assembly, we examined the binding of Syns to MTs using N-terminal His-tagged recombinant proteins. Gold-conjugated Ni-NTA particles (ϕ = 5 nm) were introduced, which bind to His tags, and then TEM was used to examine the binding patterns of Syns. No specific binding of colloidal gold particles to MTs was detected in the absence of His-tagged Syns (Supplementary Fig. S10a). Interestingly, Colloidal gold-conjugated His-αSyn (Gold-αSyn) bound to MTs and exhibited a string-like binding pattern on the MTs (Fig. 6a,b and Supplementary Fig. S10b–e). In a similar fashion, we observed string-like bindings of Gold-βSyn and Gold-γSyn to MTs (Supplementary Fig. S10f,g).

A portion of the Gold-αSyns bound by the MTs appeared to be lined up two deep and adjacent to each other in a pattern that resembled necklace-like binding around the MT (Fig. 6b and Supplementary Fig. S10g). This feature of the binding was confirmed by analyzing the TEM images after tilting (Supplementary Fig. S11). Tilting of the images allowed us to distinguish αSyn particles encircling the surfaces of MTs from αSyn particles that were simply localized in the same plane (Supplementary Fig. S11a). When the particles are located on the same flattened surface, tilting produces a simple compressed image of the width vertical to the tilting axis (Supplementary Fig. S11a upper panel and b), whereas if the particles are located on different surfaces, tilting causes deformation of the visual alignment (Supplementary Fig. S11a lower panel and c,d). Viewed in this manner, Gold-αSyn exhibited two binding patterns to MTs: simply localized in the same plane of the MTs (Fig. 6a, and Supplementary Fig. S10d) and encircling the MTs (Fig. 6b, and Supplementary Fig. S10e). The distance between the centers of the two adjacent gold particles participating in string-like binding was 7.0 ± 1.4 nm (Mean ± SD, N = 81), a value that is close to the accepted value for the separation of adjacent pfs of MT (approximately 7 nm). This string-necklace pattern was distributed intermittently on the MTs, thus imparting an appearance similar to that of bamboo joints (Supplementary Fig. S10b). We next investigated whether the binding pattern of Syn to MTs varied with the Syn concentration. At a αSyn concentration of 1 μM, oligomeric string-like bindings to MTs occurred less frequently than that at a αSyn concentration of 5 μM (Supplementary Fig. S10a,c), and an overall sparse distribution was not observed, suggesting that MTs provide a scaffold for Syn oligomerization. TEM analyses suggested that the Syns stabilize MTs by acting as hoops around a barrel, thus protecting MTs from depolymerization. We previously reported that mNudC interacts with kinesin-1 and is required for kinesin-1-mediated anterograde transport of cytoplasmic dynein complexes^16^. To explore whether αSyn promotes the binding of mNudC to MTs, we evaluated the co-localization of αSyn and mNudC on MTs. Halo-tagged αSyn (Halo-αSyn) was used to increase the intensity of the signal and provide sufficient electron density to detect the binding of αSyn without the use of gold particles. As expected, Halo-αSyn densities were clearly observable by TEM (Fig. 6c,d and Supplementary Fig. S12a,b), and they showed the same localization pattern as the Gold-αSyn particles (Supplementary Fig. S10b–e). We also observed Halo-αSyn and Gold-αSyn intensities in the border zone between the MT cylinder and the tubulin sheet (Fig. 6d,e and Supplementary Fig. S12b, blue arrowheads), where they apparently forming a hoop to promote and stabilizes MT cylinder formation. We next investigated the relevance of the αSyn and mNudC interaction using a MT pull-down assay and found that αSyn clearly enhanced the binding of mNudC to MTs (Fig. 6f). Co-localization of Gold-αSyn with Gold-anti-mNudC anbody conjugated mNudC on MT was also detected (Fig. 6g,h and Supplementary Fig. S12c–e). Our TEM results indicate that αSyn forms a scaffold apparatus on MTs, allows for binding of the kinesin adaptor protein mNudC.

### Differential interaction of αSyn with tMTs and regular MTs

To confirm the structural features of Halo-αSyn-bound MTs, samples were embedded into a thin layer of vitrified ice and observed via cryo-TEM. Consistent with the results obtained by negative staining, intensities corresponding to Halo-αSyn were observed as transverse electron-dense bands on MTs (Fig. 6i and Supplementary Fig. S12f). To determine the number of pfs in the MTs, cryo-TEM images of αSyn-bound MTs were analyzed based on the characteristic dark fringe patterns (Moiré patterns) on MTs that appear in vitreous ice^4^. MTs polymerized at 40 μM of concentrations of tubulin exhibited pfs numbers of 13–15, which shifted to 14-pfs after the addition of αSyn or Halo-αSyn (Fig. 6i,j). To our surprise, the majority of MTs that polymerized at 5 μM tubulin in the presence of αSyn contained 14-pfs, suggesting that αSyn facilitates the nucleation of 14-pfs MTs (Fig. 6j,k and Supplementary Fig. S12g). The pfs number of αSyn-bound MTs was also confirmed using axoneme- and GMPCPP-nucleated MTs. Axonemes extend >90% of 13-pfs MTs from the A-tubule and the central pair^4^. However, GMPCPP is a slowly hydrolyzable analog of GTP, and it is known to yield MT preparations in which 96% of the MTs composed of 14-pfs^52^. We examined the binding preference of TMR-Halo-αSyn to axoneme-MTs and GMPCPP-MTs^43^. Consistently, TMR-Halo-αSyn signals were preferentially detected on the GMPCPP-MTs but not on the axoneme-MTs (Fig. 6l and Supplementary Fig. S13a), which supported the idea that αSyn has a significant preference for 14-pfs MTs. We also calculated the dissociation constant for the binding of αSyn to GMPCPP-MTs (K_d_ = 0.15 μM, B_max_ = 0.83, Supplementary Fig. 14b), which is similar to the dissociation constants for the binding of cytoplasmic dynein (K_d_ ≈ 0.2 μM)^53^ and kinesin-1 (K_d_ = 0.27 μM) to MT^54^. Our findings indicate that αSyn is involved in tubulin nucleation and facilitates the polymerization of MTs containing 14-pfs.

**Figure 7:**
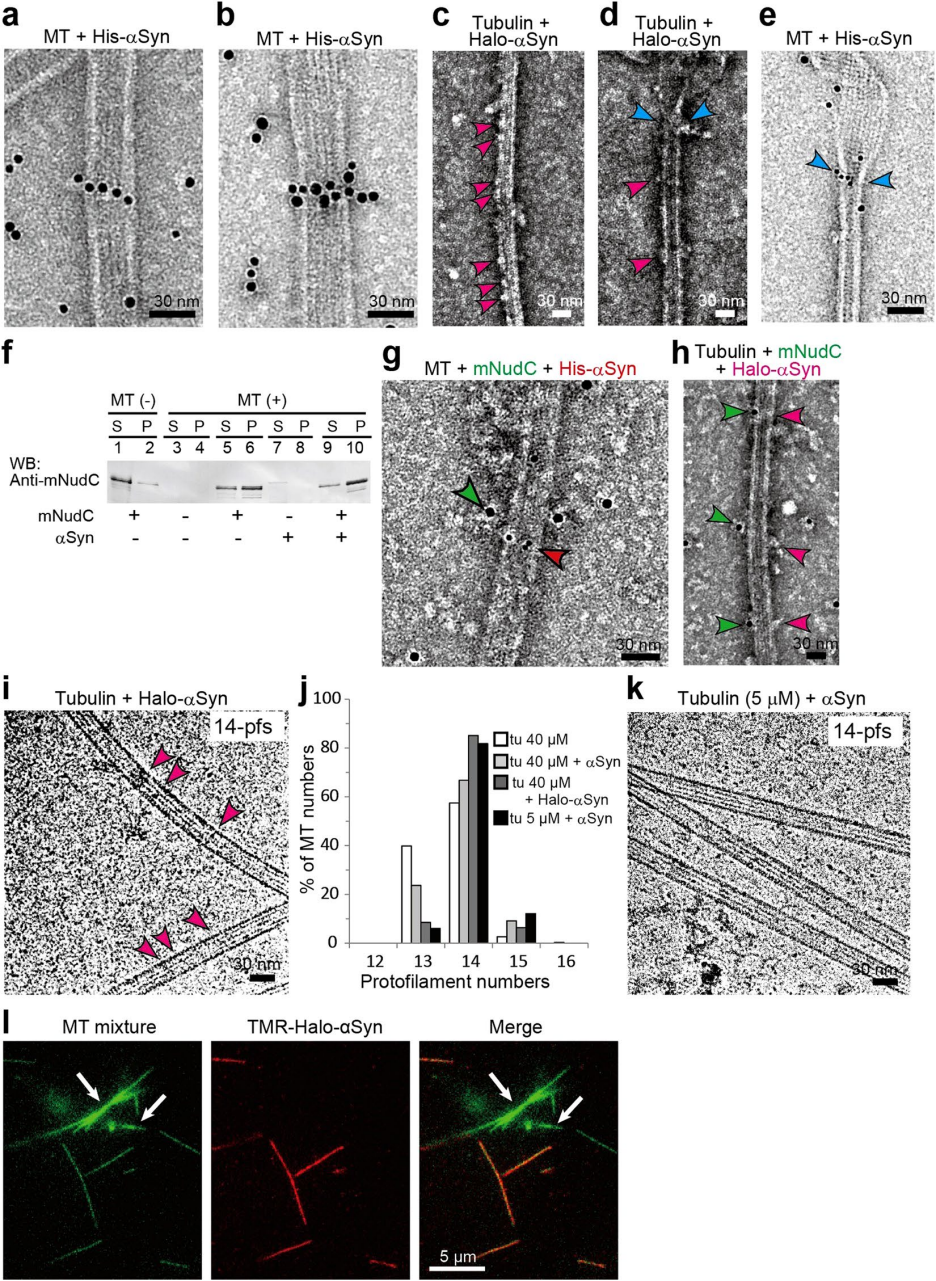
Unconventional MTs carrying 14-pfs in rat femoral nerves. Rat femoral nerves with or without ligation were embedded into resin block and examined by TEM. (**a**) Overview of a micrograph with conventional MTs carrying 13-pfs. The rectangle surrounded area was enlarged and shown in (**d**). (**b**) Overview of a micrograph showing unconventional MTs containing 14-pfs. The rectangle surrounded area was enlarged and shown in (**e**). (**c**) Overview image of the ligated femoral nerve. Unconventional MTs were captured in ligated nerve, and the rectangle surrounded area was enlarged and shown in (**f**). (**g**) Comparison of unconventional MTs in unligated and ligated femoral nerves. The percentage of MTs with 14-pfs in the unligated femoral nerve is 0.5% (10 of 2016 MTs), in the ligated nerve is 8.8% (22 of 230 MTs). (**h**) Localization of αSyn in femoral nerves visualized by IEM. Silver-enhanced gold particles are observed surrounding fuzzy material around MTs with 14-pfs (right panel), but are not visible in MTs with 13-pfs (left panel). (**i**) Model for the tMT in the anterograde transport of cytoplasmic dynein by kinesin-1. LIS1 anchors cytoplasmic dynein to a Syn-stabilized tMT followed by the tethering to a kinesin molecule under mNudC mediation. Scale bar: 50 nm in (**a**–**c**); and 25 nm in (**h**).

Although many of the cytoskeletal MTs in mammalian cells are known to contain 13-pfs^6^, our findings suggest that a specific population of MTs composed of 14-pfs may also exist in mammalian cells. To explore whether MTs carrying 14-pfs occur in mammalian cells, we examined rat femoral nerves using TEM. The femoral nerve is the largest nerve in the lumbar plexus, and it includes cable-like bundles of axons that enclose a uniform arrangement of MTs. Consistent with the results of previous work^6^, the majority of examined MTs had 13-pfs (Fig. 7a,d). Interestingly, we also found MTs with 14-pfs in both unligated and ligated femoral nerves (Fig. 7b,c,e,f and Supplementary Fig. S14). Importantly, ligation of the femoral nerve increased the number of MTs with 14-pfs to approximately 19 fold the number observed in the control (Fig. 7g). Furthermore, we detected *in vivo* interaction of αSyn with MTs carrying 14-pfs by immunoelectron microscopy (Fig. 7h). These results allow us to speculate that intrinsic MTs carrying 14-pfs are mobile and suggest that they are a strong candidate for tMTs.

## Discussion

Through this research, we discovered that Syns are required for the function of tMTs, which are associated with the anterograde transport of cytoplasmic dynein. αSyn facilitates the formation of short MTs and prevent their spontaneous depolymerization *in vitro*. In particular, αSyn preferentially binds to, and potentially helps to create, MTs carrying 14-pfs in a unique manner characterized by a ‘necklace-like’ binding of αSyn to MTs. In addition, we demonstrated the presence of mobile MTs with 14-pfs in rat femoral nerves, and these MTs are likely to be tMTs. Our findings suggest that αSyn plays an important role in the axonal transport.

MTs oscillate between phases of growth and shrinkage, and these phases are controlled by nucleotides bound to β-tubulin^1^. Because depolymerization occurs exclusively at the filament ends, GTP-tubulin caps at the plus ends of GDP-tubulin-containing MTs are believed to stabilize the growth phase. The loss of GTP-tubulin caps results in rapid shrinkage and catastrophe, with the pfs fraying or bending away from the MT axis^55^. Presumably, Syns form molecular hoops on MTs that protect against the loss of the cap and thus prevent pfs from fraying or bending away, thereby stabilizing short MT fragments. However, other MAPs, including tau protein, have not been excluded as essential components of tMTs. Indeed, live-cell imaging of mChe-tau in DRG neurons showed the presence of mobile MT fragments with cytoskeletal background. Previous studies have reported that tau binds MTs carrying 14-pfs and 13-pfs^41^, whereas DCX preferentially binds to cytoskeletal MTs that have 13-pfs^42,43^. Accordingly, mobile mChe-tau particles bind MTs with 14-pfs. More importantly, αSyn is mobile in the axon, and selectively bound to GMPCPP-nucleated 14-pfs MTs *in vitro*, suggesting that αSyn produced short MTs are mobile 14-pfs MTs, and reminiscent of tMTs. A SR-PALM analysis further revealed that endogenous αSyn associates with a filamentous structure that is sat on cytoskeletal MTs. In addition, filamentous αSyn was shown to co-localize with βIII-tubulin and DIC1. Moreover, live-cell imaging analyses showed co-migration of αSyn with βIII-tubulin and DIC1 in the anterograde transport. Our findings strongly indicate that αSyn bound mobile tMTs intimately associated with anterograde transport of cytoplasmic dynein.

tMTs have evoked intense interest and controversy for 30 years. Cytoskeletal polymers are known to move within axons, and that many cytosolic proteins are transported by binding to these cytoskeletal polymers. Previous reports have indicated that nearly 80% of the dynein that moves in an anterograde direction is associated with slowly transported MTs^17,18^. In contrast, Wang and Brown reported that these MTs are transported by fast motors^19^, which is consistent with our previous findings^15,16^. Indeed, the majority of anterogradely transported of cytoplasmic dynein is accompanied by the co-migration of tubulin^15^. We demonstrated that Syn-bound tMTs are stable short fragments with high mobility. tMTs act as specialized carriers for cytosolic proteins during motor protein-mediated transport, rather than as components of cytoskeletal MTs or passively fragmented MTs. Presumably, posttranslational modifications and MAPs, including Syns, are intimately related to the appearance of MTs with specialized cellular functions.

How are αSyn mutations implicated in the neuronal degeneration associated with PD? The most widely accepted paradigm postulates that pre-fibrillar oligomers as opposed to mature fibrils, represent neurotoxic entities in PD^32,56^. A further important insight was gained in this study through the analysis of defective αSyn transport, which was investigated in DRG neurons after the introduction of mutations in the αSyn protein. αSyn E46K and A30P notably lost their affinities for tubulin. However, αSyn E46K was associated with a more severe transport defect than αSyn A30P in DRG neurons, which resulted in a greater abnormal accumulation of αSyn in the soma. Notably, αSyn E46K has been implicated in autosomal dominant severe PD^31^, and patients harboring the E46K mutation exhibited more severe dementia^31^, which was not observed in those carrying the A30P mutation^30^. This correlation of clinical symptoms with cell biological features suggests that the contribution of αSyn to intracellular transport may be a critical factor in the pathogenesis of PD.

Eguchi *et al*. revealed that αSyn inhibits vesicle endocytosis via MT overassembly^26^. However, Vargas *et al*. showed Syns involved in early steps of synaptic vesicle endocytosis via membrane bending, and this function was defective in Syns null neurons^57^. On the other hand, Cartelli *et al*. demonstrated αSyn promotes microtubule nucleation and to enhance microtubule growth rate and catastrophe frequency, both *in vitro* and in cell^58^. Consistently, we also found αSyn involves in MT nucleation. Furthermore, αSyn facilitates to form short MTs and prevents their spontaneous depolymerization *in vitro*. These findings imply that MT dynamics regulated by Syns may linked to synaptic vesicle endocytosis, but the underlying mechanisms remain to be investigated.

Importantly, bi-directional axonal transports were severely affected by the siRNA-mediated depletion of αSyn and γSyn. In addition, the αSyn proteins with the A30P, E46K and S129E mutations exhibited abnormal accumulations in the soma and were associated with extremely reduced numbers of mobile particles, thus reinforcing the importance of αSyn in the axonal transport. Our findings also suggest that the binding potential of αSyn for MTs is intimately related to the regulation of local αSyn distribution. In other words, binding of αSyn to MTs may prevent the aberrant accumulation of αSyn. Loss of this binding potential would result in biased distribution, thereby facilitating the formation of pre-fibrillar oligomers and the cytoplasmic aggregation of αSyn, a common pathological hallmark of PD. Genomic multiplication of αSyn causes the over production of αSyn that exceeds the capacity of αSyn clearance by tMTs, thereby resulting in the aberrant accumulation of αSyn. This aberrant accumulation will leads to a PD pathogenesis.

αSyn-bound MTs carrying 14-pfs were also confirmed via the cryo-TEM analysis, which provides the first evidence for a distinctive function of MTs with unconventional numbers of pfs. Although *in vitro* tubulin polymerization yields MTs that consist of 10–15-pfs, the MTs in mammalian cells were previously thought to exclusively contain 13-pfs^6^. Remarkably, each pfs in a 13-pfs MT is arranged in a longitudinally straight manner, which is believed to be beneficial for kinesin-1 trafficking. In contrast, MTs carrying 14-pfs present a helical arrangement^59^ that is unsuitable for kinesin-1 movement. However, tMTs would not be expected to function in the same way as regular cytoskeletal MTs, because they are too small to provide a trafficking rail for motor proteins. Rather, tMTs may provide a transport platform to the periphery for proteins such as cytoplasmic dynein and the dynactin complex for which a helical pfs arrangement is not disadvantageous. It is worth exploring the distinctive functions of unconventional MTs through the identification of MAPs that can recognize MTs with unconventional numbers of pfs.

Our findings provide new insight into the regulation of the tMT-mediated transport pathway, as well as in the pathogenesis of PD. Further analysis of the generation of 14-pfs MTs and regulation of αSyn binding to MTs will provide additional insight into the pathogenesis of PD.

## Methods

The experiments performed in this study were conducted in accordance with approved guidelines. All experimental protocols were approved by the Osaka City University Graduate School of Medicine, and separate approval was provided for the recombinant DNA experiments (OCU#500) and animal experiments (OCU#08033).

### Femoral nerve ligation

Adult male Sprague–Dawley rats (200–350 g) were anesthetized via the inhalation of 4% isoflurane and then the intraperitoneal injection of pentobarbital sodium (50 μg/g body weight) prior to surgery. Both sides of the femoral nerve root were surgically exposed and then tied with a 3–0 non-absorbable silk suture, which was followed by wound closure. After 6 h of ligation, the portion of the ligated femoral nerve within 3 mm of either side of the ligation point was excised and subjected to protein extraction using PBS (137 mM NaCl, 2.7 mM KCl, 10 mM Na_2_HPO_4_ 1.8 mM KH_2_PO_4_).

### Plasmids and recombinant and purified proteins

αSyn, αSyn A30P, αSyn E46K, αSyn S129A, αSyn S129E, βSyn, γSyn, tau, DIC1 and DCX were subcloned into pmCherry-C1 (Clontech Laboratories, CA, USA); VP26 was subcloned into the pmCherry-N1 vector; tau, DIC1, LIS1 and mNudC were subcloned into pmNeonGreen; and β III-tubulin was subcloned into the pmTurquoise2 vector.

For the lentivirus-mediated expression system, HIV-1-based lentiviral vectors pseudotyped with the vesicular stomatitis virus G-glycoprotein (VSV-G) were generated by transient transfection of 293 T cells with three plasmids, the packaging construct (pCAG-HIVgp), the VSV-G and Rev expressing construct (pCMV-VSV-G-RSV-Rev), and the self-inactivating lentiviral vector construct (pCSII-EF-MCS-IRES2). These materials were kindly provided by Dr. Atsushi Miyawaki of the RIKEN Brain Science Institute, Japan. Transfection of plasmids and collection of lentivirus were performed using the recommended methods (http://cfm.brc.riken.jp/lentiviral-vectors/protocols/). After removing the Venus label, each targeting cDNA fragment fused with mCherry (mChe), mNeonGreen (mNG) or mTurquoise2 (mTQ) tag as described above was subcloned into the pCSII-EF-MCS-IRES2 vector.

GST-conjugated recombinant αSyn, βSyn, γSyn, Halo-αSyn, His-αSyn and mNudC were generated using a bacterial expression system (Thermo Fisher Scientific, MA, USA) and the pGEX-4T expression vector (GE Healthcare Lifesciences, UK). Recombinant protein purification was performed using GST-Sepharose (GE Healthcare, UK) according to the manufacturer’s instructions. The GST tag was removed from recombinant proteins via thrombin proteolytic cleavage (GE Healthcare Lifesciences, UK) or with PreScission protease (GE Healthcare Lifesciences, UK). Tubulin purified from porcine brain was separated from the MAPs via phosphocellulose (P-11; Whatman, UK) chromatography, and polymerized MTs were prepared using assembly and disassembly cycles in BRB80 buffer containing 1 mM GTP.

### Immunoprecipitation

For the immunoprecipitation assay, anti-βIII-tubulin antibody (MAB1195; R&D Systems, USA) was biotinylated with Biotin-(AC5)2 Sulfo-OSu (341–06801; Dojindo, Japan) and mixed with streptavidin-linked agarose (69203; Novagen, USA) for 2 h at room temperature followed by three washes in chilled BRB80 buffer (80 mM Pipes–KOH, pH 6.8, 2 mM MgSO_4_, and 1 mM EGTA). Femoral nerve lysates (0.5 mg of protein) were then added, and the mixture was gently agitated for 2 h at 4 °C. After three washes, co-precipitates were eluted with 0.1 M glycine–HCl (pH 2.8) for 30 min. The eluted material was neutralized with 1 M Tris (approximate pH 10) and subjected to subsequent proteomic analysis or silver staining.

### Isolation and proteomic analysis of tubulin client proteins

For the MS analysis, the eluted and neutralized immunoprecipitates were subjected to desalting and concentration by SDS-PAGE on a 5%–20% polyacrylamide gradient gel (Wako, Japan), followed by staining with Quick-CBB (Wako, Japan). Gel slices of approximately 1-mm thickness per lane were excised and destained in 25 mM NH_4_HCO_3_ and 50% acetonitrile followed by an additional destaining step in 25 mM NH_4_HCO_3_ and 30% acetonitrile and reduction in 25 mM NH_4_HCO_3_ containing 10 mM DTT at 56 °C for 1 h. The reduced cysteine residues were alkylated with 55 mM iodoacetamide in 25 mM NH_4_HCO_3_, and the gel pieces were washed in acetonitrile and dried. The dried gel pieces were treated with 10 µg/ml trypsin (Trypsin Gold; Promega, USA) in 50 mM NH_4_HCO_3_ on ice for 30 min. Excess trypsin was then removed, and complete digestion was allowed to occur at 37 °C for 18 h in 50 mM NH_4_HCO_3_. The samples were desalted using Zip Tip C18 (Millipore, USA) in accordance with the manufacturer’s instructions. Trifluoroacetic acid was then added to a final concentration of 0.1% and the material was used for a nano-LC-electrospray ionization-MS/MS (LC-ESI-MS/MS) analysis.

### Nano-ESI-LC-MS/MS analysis and protein identification

The LC-MS/MS analysis of immunoprecipitates was performed on a DiNa-AI nano LC system (KYA Technologies, Japan) coupled to a QSTAR Elite hybrid mass spectrometer (AB Sciex, Canada) through a NanoSpray ion source (AB Sciex). The data acquisition was performed using Analyst QS Software 2.0 (AB Sciex) in the positive-ion mode. Both sets of data were processed using ProteinPilot with the Paragon™ search algorithm (AB Sciex). The MS/MS data were used as an NCBI database search query. The minimum threshold for protein identification was set at a protein score of 0.47, which corresponded to a confidence level of >66% with a false discovery rate of 1%.

### Generation of an antibody against γSyn

To prepare an anti-γSyn antibody, we immunized New Zealand white rabbits with GST-conjugated recombinant γSyn that had been expressed in bacteria and purified using GST-Sepharose (GE Healthcare, UK) according to standard procedures. The antiserum was collected and used in the experiments (Supplementary Fig. S1b).

### Western blotting (WB) analysis

Cells and tissues were lysed in lysis buffer. For immunoblotting, proteins were separated using SDS-PAGE under reducing conditions, which was followed by electrophoretic transfer to PVDF membranes. The membranes were probed using antibodies against β-actin (1:1000, A2228; Sigma-Aldrich, USA), βIII-tubulin (1:1000, MAB1195, R&D Systems, USA) αSyn (1:500, 610787; BD Biosciences, USA), and βSyn (1:1000, ab76111; Abcam, UK) as well as antiserum raised against γSyn at 1:500 dilution followed by incubation with a secondary antibody conjugated to alkaline phosphatase or horseradish peroxidase at 1:1000 dilution. The blots were developed using the BCIP/NBT phosphatase substrate system (11 681 451 001; Roche, Switzerland) or ECL Prime Western Blotting Detection reagent (GE Healthcare Bio-Science, UK). To detect αSyn phosphorylation, tissues were homogenized in freshly prepared lysis buffer containing a protease inhibitor cocktail. The membrane was probed using an antibody specific for αSyn protein phosphorylated at serine 129 (1:500, ab42906, Abcam, UK).

### Immunocytochemistry

After 24 h of culture, cells were fixed in 4% (w/v) ultrapure electron microscopy-grade paraformaldehyde (PFA) for 15 min and permeabilized using 0.2% Triton X-100 for 5 min at room temperature. The cells were then blocked using 3% (w/v) BSA in PBS and incubated with antibodies against αSyn (1:250), βSyn (1:500), γSyn (1:250), βIII-tubulin (1:1000), and Tau-1 (1:1000, MAB3420, Chemicon, USA) followed by incubation with Alexa 488-conjugated secondary antibodies (1:1000, Molecular Probes, USA). Nuclei were probed with 100 nM 4′,6-diamidino-2-phenylindole (DAPI) (D523, Dojindo, Japan). Each incubation was performed for 1 h at room temperature. Slides were then mounted with FluorSave Reagent (345789, Calbiochem, USA).

### Live-cell one-color imaging

DRG neurons were prepared as described in Supplementary methods. Immediately after dissection, DRGs were electroporated with expression vectors for various proteins using a Neon transfection system or lentivirus-mediated expression system. Live-cell imaging was performed 24–48 h after transfection or infection. An IX70 inverted microscope (Olympus, Japan) equipped with an incubator at 37 °C (MATS-LH; Tokai Hit, Japan) was used to track mCherry-fused target proteins in the axons of DRG neurons. Images were captured using a digital charge-coupled device (CCD) camera (EM-CCD C9100-13; Hamamatsu Photonics K.K., Japan) and recorded movies were analyzed using previously described custom software^60^. The centroid of the particle in each image was determined by fitting the images to 2D-Gaussian curves.

### Live-cell triple-color imaging

mTQ-βIII-tubulin and mCherry-αSyn were co-expressed with mNG-DIC1, mNG-LIS1 or mNG-mNudC in DRGs using the Neon transfection system and excited sequentially using a light-emitting diode (LED) (SPECTRA X light engine, Lumencor, OR, USA) passed through each excitation filter (FF01-427/10 for mTQ, FF01-504/12 for mNG, and FF01-589/15 for mCherry; Semrock Inc., IL, USA). Fluorescence images were obtained using an inverted microscope (Ti-E, Nikon Instech Co., Tokyo, Japan) with an oil-immersion objective lens (Plan Apo 60x, numerical aperture 1.40, Nikon Instech Co., Tokyo, Japan). Each emission was split 3 ways using a dichroic mirror (FF444-521-608-Di01, Semrock Inc., USA). The images were passed through an emission filter (FF01-465/30 for mTQ, FF01-537/26 for mNG, and FF01-628/40 for mCherry; Semrock Inc., USA) with a filter wheel system (99A351, Ludl Electronic Products Ltd., Hawthorne, NY, USA) and captured simultaneously with an electron-multiplying CCD camera (DU-897U iXon Ultra; Andor Technology, Belfast, Northern Ireland). All experiments were performed at a temperature of 37 °C maintained using a lens heater (Tokai-Hit, Shizuoka, Japan). For single particle tracking of mTQ, mNG, and mCherry fusion proteins, the axonal regions of DRGs were observed continuously. Time-lapse images at 100-ms exposure were acquired at 120 time points separated by 500-ms intervals and further processed using ImageJ software (National Institutes of Health, Maryland, USA) and MetaMorph software (Molecular Devices, LLC, CA, USA).

### SR-SIM

mTQ-βIII-tubulin, mNG-DIC1, mNG-LIS1, mNG-mNudC, and mCherry-αSyn were co-expressed in DRGs as described above; subsequently, the DRGs were fixed with 4% PFA and excited sequentially using 458-, 488-, and 561-nm lasers (Sapphire; Coherent, CA, USA). Fluorescence images were obtained using a structured illumination microscope with a 3-dimensional illumination pattern (N-SIM; Nikon Instech Co., Japan) through a water-immersion objective lens (Plan Apo 60x, numerical aperture 1.27; Nikon Instech Co., Japan). The axonal regions of the DRGs were observed for single particle viewing of the mTQ, mNG, and mCherry fusion proteins. The images were acquired with 500-ms exposures and further processed using ImageJ and MetaMorph software.

### siRNA preparation and transfection

Deprotected double-stranded 21-nucleotide RNAs targeting mouse αSyn and γSyn sequences were synthesized by Sigma-Aldrich, Japan, and the product codes were Mm_Snca_0386_s and Mm_sncg_5455_s, respectively. Firefly (*Photinus pyralis*) luciferase siRNA served as the negative control. siRNAs were transfected into DRG neurons using the Neon transfection system.

### Dark-field microscopic imaging of MTs

MAP-depleted tubulin (40 µM) was mixed with Syn (20 µM) in BRB80 buffer containing 1 mM GTP and incubated for 30 min at 37 °C. After incubation, the mixture was stabilized by the addition of 10 µM paclitaxel (T1912, Sigma-Aldrich, USA) and diluted 200 fold. MTs were observed using dark-field illumination and a 40× objective lens. The images were captured with a digital CCD camera (excel-V; Dage-MTI, Michigan, USA). The MTs in each visual field were identified, and their lengths were measured. Approximately 80 MTs were measured to determine the average MT length for each condition.

To observe MT depolymerization, biotinylated tubulin (4 mg/ml) was mixed with Syn (20 µM) in BRB80 buffer containing 1 mM GTP and incubated for 30 min at 37 °C. After incubation, the mixture was stabilized by the addition of 1 µM paclitaxel and diluted 200 fold. We used untreated glass slides (#1 glass slide, 26 mm × 76 mm, S-1126; Matsunami, Osaka, Japan) as the observation flow chambers. A total of 10 µl of 0.5 mg/ml streptavidin was applied to the chamber and absorbed onto the glass for 10 min. The following were then added in sequence: (i) 2 volumes of BRB80 buffer to wash out excess streptavidin, (ii) 1 volume of biotinylated MTs containing 1 µM paclitaxel, and (iii) 2 volumes of BRB80 buffer to wash out unattached MTs. The MTs subsequently began to depolymerize, and this process was observed using dark-field illumination and a 40 × objective lens. The recorded spontaneous MT depolymerizations were analyzed as described above. MTs attached to the glass surface in the visual field were identified, and tip displacement was measured.

### Negative staining for TEM

Purified tubulin and Syns were mixed at a molar ratio of 1:3, diluted with BRB80 buffer to the desired tubulin concentration (5–50 µM), and incubated with 1 mM GTP at 37 °C for 30 min. MTs were stabilized with paclitaxel after incubation. To observe negatively stained specimens, 5 µl of 0.5 µM paclitaxel-stabilized MTs were loaded onto a hydrophilized carbon grid (300 mesh; Cu, VECO, USA) and incubated for 60 s. The specimen was subsequently washed 3 times in paclitaxel buffer to remove the excess solution (including free proteins) and stained with 2% uranyl acetate for 40 s. For the imaging of Syn-bound MTs, purified His-Syn was labeled with 5 nm Ni-NTA-gold (2082; Nanoprobes, USA) at 4 °C for 1 h. paclitaxel-stabilized MTs and gold-labeled Syn were mixed at a molar ratio of 1:1 and incubated for 30 min at room temperature to form MT-Syn complexes. mNudC (3 μM) was incubated with anti-mNudC antiserum for 1 h and subsequently incubated with secondary antibodies conjugated with 10-nm colloidal gold (EMGAR10, BB International, UK) for 1 h.

The specimens were observed under a TEM (Tecnai Spirit, FEI, USA) at 120 kV at a nominal magnification of 64,000 × (3.23 Å/pixel) and under-focus values of 1–1.5 µm. Images were recorded using a CCD camera (2 k × 2 k Eagle^TM^1k CCD, 30 µm/pixel corresponding to 0.47 nm; FEI). TEM image processing was performed using the Extensible object-oriented system (Eos) and ImageJ as described in Supplementary methods.

### *In vitro* MT imaging assay

To characterize the binding of αSyn to MTs, 13-pfs and 14-pfs MTs were polymerized from axonemes and GMPCPP, respectively. *Chlamydomonas reinhardtii* (*C. reinhardtii*) axoneme and purified tubulin were used as previously described^43^. *C. reinhardtii* flagella were collected and demembranated with 0.2% NP-40/BRB80 for 5 min on ice followed by washing three times. To polymerize MTs carrying 13-protofilaments (pfs), approximately 1–2 mg/ml axonemes were incubated with ATTO488 (Funakoshi, Japan) labeled tubulin (ATTO488-tubulin) and 10 mM GTP for 20 min at 37 °C. After incubation, the MTs were stabilized by the addition of 10 μM paclitaxel. MTs carrying 14-pfs were polymerized by incubating of ATTO488-tubulin and 1 mM GMPCPP (NU-405S, Jena Bioscience) together at 37 °C for 1 h and then stabilized with 10 μM paclitaxel. For the observations, anti-α-tubulin antibody (TU-02, sc-8035, Santa Cruz Biotechnology Inc., Texas, USA) was introduced into a flow chamber and allowed to remain for 10 min followed by the addition of 5 mg/ml casein solution for blocking. After attaching the flow chamber to a microscope equipped with an objective lens heater set at 37 °C, MTs carrying 13- and 14-pfs were mixed in BRB80/10 μM paclitaxel buffer, and introduced into the flow chamber. TMR-αSyn was prepared with recombinant Halo-αSyn and HaloTag TMR Ligand (Promega, USA) in according to the manufacturer’s protocol. A solution of 0.5 μM TMR-αSyn in imaging buffer (BRB80, 10 μM paclitaxel, 1 mM DTT, 1 M glucose, 3.6 mg/ml catalase, 5 mg/ml glucose oxidase) was introduced into the flow chamber, and images were recorded using a CCD camera (EM-CCD C9100-13; Hamamatsu Photonics K.K., Japan).

### Steady-state MT binding assay

MTs were polymerized for 30 min in the presence of 10 mM GTP or 1 mM GMPCPP (NU-405S, Jena Bioscience). A steady-state binding assay was performed by co-sedimentation of αSyn with MT in BRB80 buffer containing 10 μM paclitaxel at 25 °C. In the experiments, 0.05 μM αSyn was mixed with various concentrations (up to 8 μM) of paclitaxel-stabilized MTs. After incubation for 30 min, the mixtures were centrifuged at 100,000 × g for 10 min. The supernatants and precipitates were collected, and the concentration of the αSyn in both portions was analyzed by WB. The data for [αSyn]_bound_/[αSyn]_total_ corresponding to the reaction rate versus the substrate [MT] concentration were fitted to a rectangular hyperbola, and K_d_ and B_max_ were calculated using the Michaelis-Menten equation. Here, K_d_ corresponds to the dissociation constant and B_max_ corresponds to the maximum binding.

### Ultra-thin sections of rat femoral nerve

Femoral nerves were resected from 3-months-old rats with or without femoral nerve ligation and treated with 0.1% Triton X-100 for 2 h at room temperature followed by fixation in 1% glutaraldehyde/tannic acid. MT stabilizing buffer (4 M glycerol, 0.1 M PIPES, pH 6.8, 1 mM MgSO_4_, and 2 mM EGTA) was used during the experiment. The femoral nerves were then dehydrated through a graded ethanol series (50, 70, 80, 90 and 100%), embedded in Epon 812 resin (TAAB, UK), and then cut into ultra-thin sections at a 50 nm thickness using an Ultramicrotome EM UC-6 (Leica Microsystems, Vienna, Austria). The sections were finally stained with 0.4% lead citrate and observed under a TEM (Tecnai Spirit, FEI, USA or JEM-3100FEF, JEOL, Japan) at 120 kV or 300 kV at a nominal magnification of 110,000x (0.197 nm/pixel) or 40,000x (0.416 nm/pixel) and under-focus values of 1–1.5 µm. Images were recorded using a CCD camera (2 k × 2 k Eagle^TM^1k CCD, or 1k × 1k Gatan CCD). TEM image processing was performed using Eos as described in Supplementary methods.

### Immunoelectron microscopy

For the immunoelectron microscopy analyses, femoral nerves from rats at postnatal day 2 were treated for 20–30 min with 0.1% Triton X-100 containing 4 M glycerol/PME (0.1 M PIPES, 1 mM MgSO_4_, 2 mM EGTA, pH 6.8) and then fixed for 2 h in 2% PFA containing 1% glutaraldehyde and 10 μM paclitaxel. Following fixation, the nerves were replaced in 30% sucrose/0.1 M phosphate buffer (PB) overnight, and then they werre embedded in OCT compound (Sakura Finetek, Japan) and frozen in liquid nitrogen. The frozen sections were cut to a 12 µm thickness using a cryostat (Microm HM 560 Cryostat, Thermo Fisher Scientific, USA), and thaw mounted onto poly-L-Lysine-coated plastic coverslips (162-09311, Wako, Japan).

The sections were washed three times in PBS and permeabilized for 5 min in 0.2% triton X-100/PBS. After washing, blocking was performed for 5 min in 4% Block Ace Powder (DS Pharma Biomedical Co.,Ltd., Japan) and 0.005% saponin. Then samples were then treated with anti-αSyn antibody (1:100) for 3 h, followed by the incubation with anti-mouse Fab’ fragment conjugated to 1.4 nm Nanogold (#2004, Nanoprobes, USA) (1:20) overnight at 4 °C. Next, the samples were washed twice with HEPES buffer (0.1 M, pH 7.5) containing 0.005% saponin, and washed three times with HEPES buffer, and fixed in 2.5% glutaraldehyde and 0.5% tannic acid in HEPES buffer for 1 h. Particles much larger in size were obtained using the HQ SILVER silver enhancement kit (#2012, Nanoprobes, USA) according to the manufacturer’s protocol. The samples were then fixed in 0.5% osmium tetroxide for 30 min on ice and stained with 1% uranyl citrate for 4 h at 4 °C. The stained samples were then dehydrated and embedded into Epon 812 resin (TAAB, UK). Ultrathin sections (~50 nm thickness) were cut on an Ultramicrotome EM UC-6 (Leica Microsystems, Vienna, Austria) and stained with 0.4% lead citrate before observation. TEM image processing was performed using Eos as described in Supplementary methods.

## Electronic supplementary material

Supplementary figures

Supplementary movie 1

Supplementary movie 2

Supplementary movie 3

Supplementary movie 4

Supplementary movie 5

Supplementary movie 6

Supplementary movie 7
