# Publisher Correction: Alpha-synuclein facilitates to form short unconventional microtubules that have a unique function in the axonal transport

**DOI:** 10.1038/s41598-018-25979-4

**Published:** 2018-05-17

**Authors:** Shiori Toba, Mingyue Jin, Masami Yamada, Kanako Kumamoto, Sakiko Matsumoto, Takuo Yasunaga, Yuko Fukunaga, Atsuo Miyazawa, Sakiko Fujita, Kyoko Itoh, Shinji Fushiki, Hiroaki Kojima, Hideki Wanibuchi, Yoshiyuki Arai, Takeharu Nagai, Shinji Hirotsune

**Affiliations:** 1 Department of Genetic Disease Research, Osaka City University Graduate School of Medicine, Asahi-machi 1-4-3 Abeno, Osaka, 545-8585 Japan; 2 Department of Bioscience and Bioinformatics, Faculty of Computer Science and Systems Engineering, Kyushu Institute of Technology, Kawazu 680-4, Iizuka, Fukuoka, 820-850 Japan; 3 JST-SENTAN, 4-1-8, Honcho, Kawaguchi, Saitama, 332-0012 Japan; 4 JST-CREST, 4-1-8, Honcho, Kawaguchi, Saitama, 332-0012 Japan; 5 Graduate School of Life Science, University of Hyogo, 3-2-1 Kouto, Kamigori-cho, Ako-gun, Hyogo, 678-1297 Japan; 6 RSC-University of Hyogo Leading Program Center, RIKEN SPring-8 Center, 1-1-1 Kouto, Sayo-cho, Sayo-gun, Hyogo, 679-5148 Japan; 7 Graduate School of Materials Science, Nara Institute of Science and Technology, 8916-5, Takayama, Ikoma, Nara, 630-0101 Japan; 8 Department of Pathology and Applied Neurobiology, Kyoto Prefectural University of Medicine Graduate School of Medical Sciences, Kajii-cho, Kawaramachi-Hirokoji, Kamigyo-ku, Kyoto, 602-8566 Japan; 9 Advanced ICT Research Institute, National Institute of Information and Communications Technology, 588-2 Iwaoka, Nishi-ku, Kobe, 651-2492 Japan; 10 Department of Pathology, Osaka City University Graduate School of Medicine, Asahi-machi 1-4-3 Abeno, Osaka, 545-8586 Japan; 11 Department of Biomolecular Science and Engineering, Institute of Scientific and Industrial Research, Osaka University, Mihoga-oka 8-1, Osaka, 567-0047 Japan

Correction to: *Scientific Reports* 10.1038/s41598-017-15575-3, published online 27 November 2017

In this Article, Figure 5 was inadvertently published as Figures 5 and 6, leading to the incorrect publication of Figure 6 as Figure 7 and the omission of the correct Figure 7. The correct Figures 5, 6, and 7 appear below as Figures 1, 2, and 3 respectively. The Figure legends are correct.

**Figure 1:**
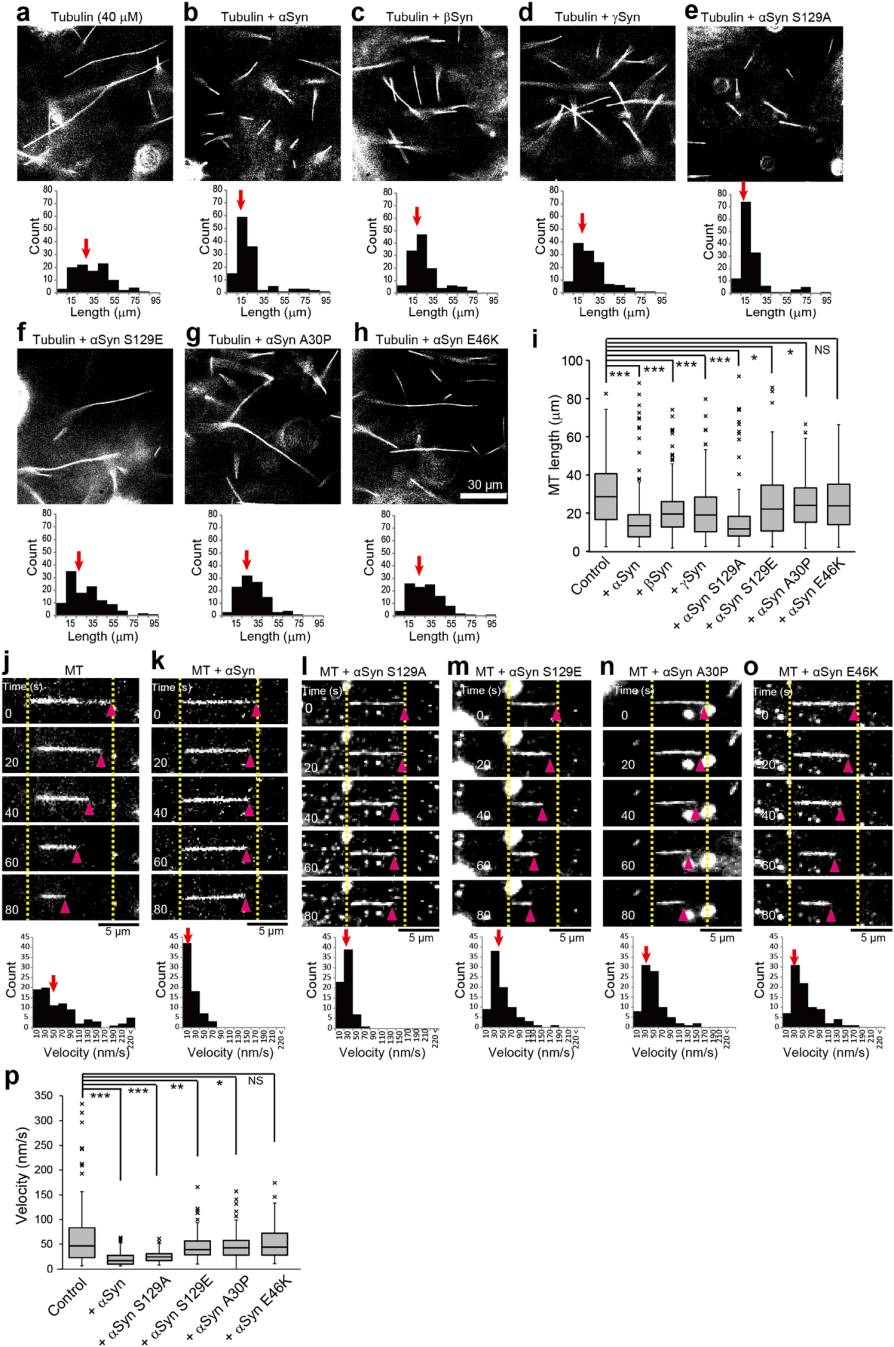
Effect of Syns on tubulin polymerization and depolymerization. (**a**–**h**) MTs undergoing polymerization with unlabeled tubulin in vitro were visualized using dark-field light microscopy. Tubulin polymerization was performed without Syns (**a**) and with αSyn (**b**), βSyn (**c**), γSyn (**d**), αSyn S129A (**e**), αSyn S129E (**f**), αSyn A30P (**g**), or αSyn E46K (**h**). MT length with or without Syns was measured and is shown beneath each image. The median values are indicated by red arrows. Scale bar: 30 μm. (**i**) Box-and-whisker plots of the lengths of MTs polymerized in vitro (N = 100 for each condition). (**j**–**o**) Effect of αSyns on MT stabilization. Unilaterally occurring spontaneous depolymerization was measured in vitro without αSyns (**j**) and with αSyn (**k**), αSyn S129A (**l**), αSyn S129E (**m**), αSyn A30P (**n**), or αSyn E46K (**o**). The distributions of the depolymerization velocities are shown beneath each image set. Spontaneous depolymerization proceeded unilaterally in each case. The pink arrowheads indicate the tips of depolymerizing MTs, and the dotted yellow lines indicate the original lengths of the MTs. The median values are indicated by red arrows. Scale bar: 5 μm. (**p**) Box-and-whisker plots of the spontaneous depolymerization velocities (N = 80 in each condition). P values in (**i**) and (**p**) were calculated with t-test of nonparametric test, mean ± SEM; ***p < 0.001, **p < 0.01, *p < 0.05, “NS” means not significant. See also Supplementary Videos 6 and 7.

**Figure 2:**
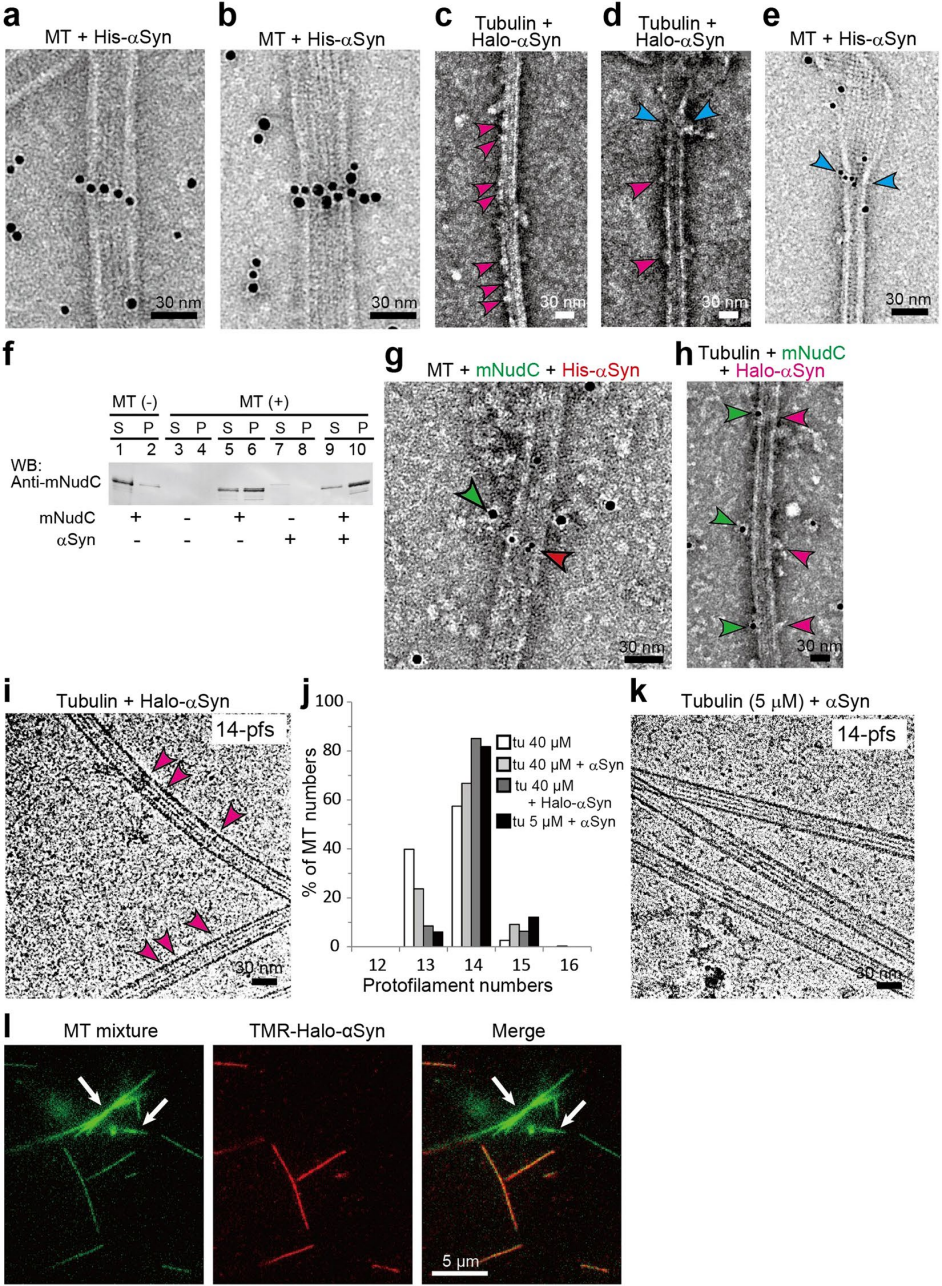
Characterization of αSyn binding to MTs using colloidal gold particles and Halo-tags. (**a**,**b**) αSyn binding to MTs was analyzed by transmission electron microscope (TEM). MTs were mixed with colloidal gold-labeled αSyn (Gold-αSyn) and negatively stained with 2% of uranyl acetate. Gold-αSyn was prepared from N-terminal His-tagged αSyn (His-αSyn). Gold-αSyns appeared as string-like αSyn polymers on MTs. (**c**) TEM image of MT polymerized with Halo-tagged αSyn (Halo-αSyn). Bamboo joint-like structures (indicated by magenta arrowheads) are visible on the MTs. (**d**) MT end structure with Halo-αSyn. Joint-like structures similar to those shown in (**c**) are indicated by magenta arrowheads. Halo-αSyns were also observed in the zone between the outwardly opened tubulin sheet and MT cylinder (blue arrowheads). (**e**) Gold-αSyn located at the transition zone (blue arrowheads). (**f**) MT pull-down assay. mNudC co-precipitated with MTs was examined in the absence and presence of αSyn. (**g**) Dual labeling immunoelectron microscopy (IEM) used to visualize the interaction of MT with mNudC and αSyn. mNudC was labeled with 10 nm colloidal gold (green) via antimNudC antibody; and His-αSyn was labeled with 5 nm colloidal gold (red). Co-localization of mNudC and αSyn on a MT is indicated by arrowheads. (**h**) MT polymerized with Halo-αSyn (magenta) and Gold-mNudC (green). Bamboo joint-like structures (magenta) and colloidal gold (green) indicate co-localization of mNudC with Halo-αSyn on a MT. (**i**) Cryo-TEM image of MTs polymerized with Halo-αSyn. Joint-like structures on MTs are indicated by magenta arrowheads. MT pfs numbers determined from Moiré patterns are indicated at the top right. (**j**) Distribution of the pfs numbers of polymerized MTs. The MTs assembled from 40 μM of tubulin (tu) without paclitaxel stabilization mainly formed 13- and 14-pfs MTs (for tu 40 μM, N = 261). The addition of αSyn clearly increased the number of MTs carrying 14-pfs even at 5 μM tubulin (tu 40 μM + αSyn, N = 259; tu 40 μM + Halo-αSyn, N = 135; tu 5 μM + αSyn, N = 111). (**k**) Cryo-TEM image of MTs polymerized with αSyn and 5 μM of tubulin. MT pfs numbers determined from Moiré patterns are indicated at the top right. Bar: 30 nm. (**l**) Selective binding of αSyn to MTs. A mixture of axoneme-nucleated MTs (axoneme-MTs) and GMPCPP polymerized MTs (GMPCPP-MTs) was incubated with TMR-Halo-αSyn (red) in a chamber. The white arrows indicate axoneme-MTs; narrow MTs correspond to GMPCPP-MTs. TMR-Halo-αSyn appears to preferentially bind to GMPCPP-MTs, but not to axoneme-MTs. Scale bar: 30 nm in (**a**–**e**), (**g**–**i**) and (**k**); and 5 μm in (**l**).

**Figure 3:**
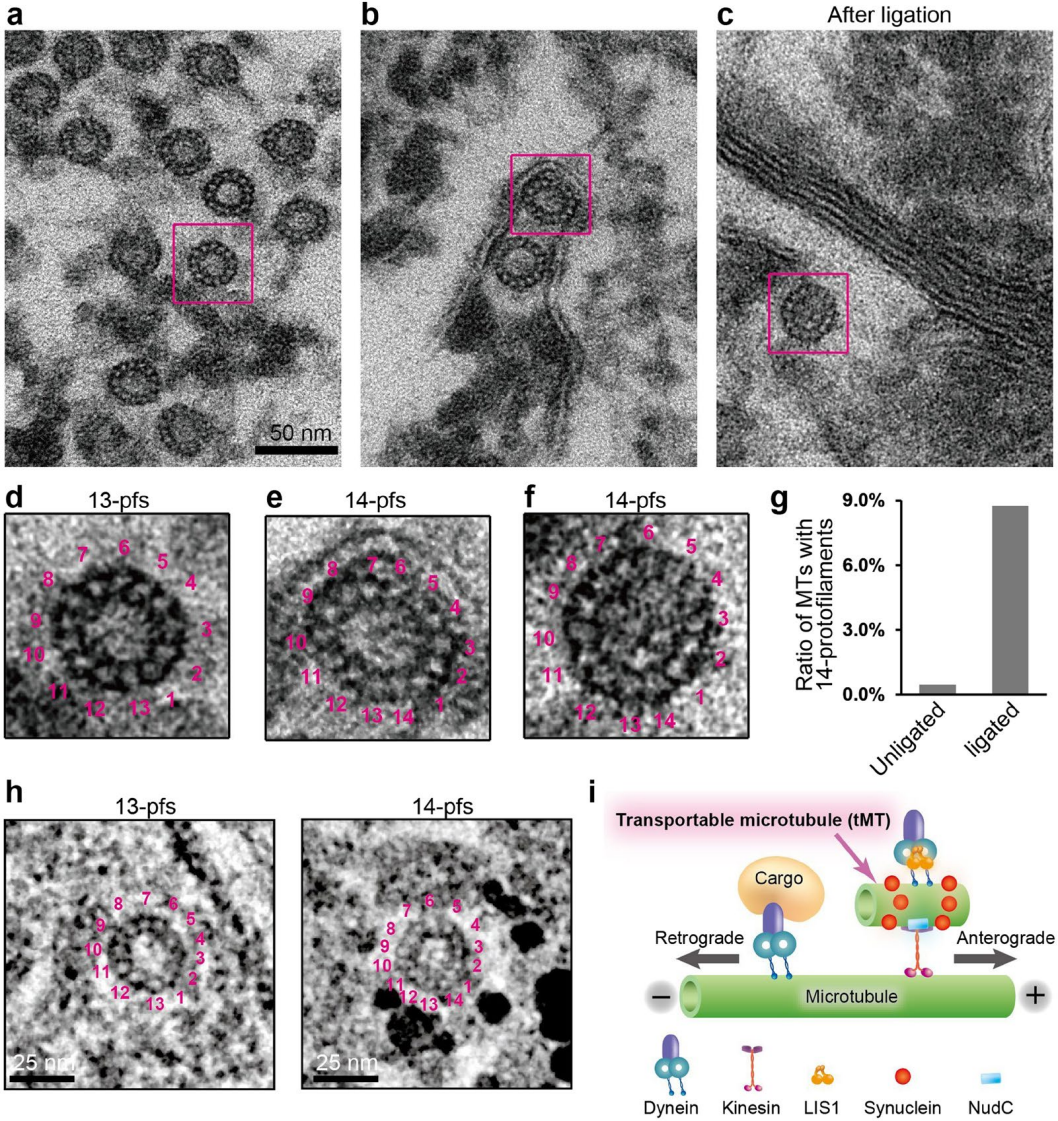
Unconventional MTs carrying 14-pfs in rat femoral nerves. Rat femoral nerves with or without ligation were embedded into resin block and examined by TEM. (**a**) Overview of a micrograph with conventional MTs carrying 13-pfs. The rectangle surrounded area was enlarged and shown in (**d**). (**b**) Overview of a micrograph showing unconventional MTs containing 14-pfs. The rectangle surrounded area was enlarged and shown in (**e**). (**c**) Overview image of the ligated femoral nerve. Unconventional MTs were captured in ligated nerve, and the rectangle surrounded area was enlarged and shown in (**f**). (**g**) Comparison of unconventional MTs in unligated and ligated femoral nerves. The percentage of MTs with 14-pfs in the unligated femoral nerve is 0.5% (10 of 2016 MTs), in the ligated nerve is 8.8% (22 of 230 MTs). (**h**) Localization of αSyn in femoral nerves visualized by IEM. Silver-enhanced gold particles are observed surrounding fuzzy material around MTs with 14-pfs (right panel), but are not visible in MTs with 13-pfs (left panel). (**i**) Model for the tMT in the anterograde transport of cytoplasmic dynein by kinesin-1. LIS1 anchors cytoplasmic dynein to a Syn-stabilized tMT followed by the tethering to a kinesin molecule under mNudC mediation. Scale bar: 50 nm in (**a**–**c**); and 25 nm in (**h**).

