# Supplementary material for: Alpha-synuclein facilitates to form short unconventional microtubules that have a unique function in the axonal transport
Source: Sci Rep. 2017 Nov 27;7:16386. doi: 10.1038/s41598-017-15575-3 (PMC5703968; doi:10.1038/s41598-017-15575-3)

Supplementary Information

Alpha-synuclein facilitates to form short unconventional microtubules that have a unique function in the axonal transport

Shiori Toba[1]†, Mingyue Jin[1]†, Masami Yamada[1], Kanako Kumamoto[1], Sakiko Matsumoto[1], Takuo Yasunaga[2,3,4], Yuko Fukunaga[5,6], Atsuo Miyazawa[5,6], Sakiko Fujita[7], Kyoko Itoh[8], Shinji Fushiki[8], Hiroaki Kojima[9], Hideki Wanibuchi[10], Yoshiyuki Arai[11], Takeharu Nagai[11] and Shinji Hirotsune[1]*

[1]Department of Genetic Disease Research, Osaka City University Graduate School of Medicine, Asahi-machi 1-4-3 Abeno, Osaka 545-8586, Japan.

[2]Department of Bioscience and Bioinformatics, Faculty of Computer Science and Systems Engineering, Kyushu Institute of Technology, Kawazu 680-4, Iizuka, Fukuoka 820-850, Japan.

[3]JST-SENTAN, 4-1-8, Honcho, Kawaguchi, Saitama 332-0012, Japan.

[4]JST-CREST, 4-1-8, Honcho, Kawaguchi, Saitama 332-0012, Japan.

[5]Graduate School of Life Science, University of Hyogo, 3-2-1 Kouto, Kamigori-cho, Ako-gun, Hyogo 678-1297, Japan.

[6]RSC-University of Hyogo Leading Program Center, RIKEN SPring-8 Center, 1-1-1 Kouto, Sayo-cho, Sayo-gun, Hyogo 679-5148, Japan.

[7]Graduate School of Materials Science, Nara Institute of Science and Technology, 8916-5, Takayama, Ikoma, Nara 630-0101, Japan.

[8]Department of Pathology and Applied Neurobiology, Kyoto Prefectural University of Medicine Graduate School of Medical Sciences, Kajii-cho, Kawaramachi-Hirokoji, Kamigyo-ku, Kyoto 602-8566, Japan.

[9]Advanced ICT Research Institute, National Institute of Information and Communications Technology, 588-2 Iwaoka, Nishi-ku, Kobe 651-2492, Japan.

[10]Department of Pathology, Osaka City University Graduate School of Medicine, Asahi-machi 1-4-3 Abeno, Osaka 545-8586, Japan.

[11]Department of Biomolecular Science and Engineering, Institute of Scientific and Industrial Research, Osaka University, Mihoga-oka 8-1, Osaka 567-0047, Japan.

†These authors contributed equally to this work.

Correspondence and requests for materials should be addressed to S.H. (email: shinjih@med.osaka-cu.ac.jp)

Dr. Yamada's present address: Department of Cell Biology & Biochemistry, Division of Medicine, Faculty of Medical Sciences, University of Fukui, 23-3 Shimoaizuki Matsuoka, Eiheiji-chou, Yoshida-gun, Fukui 910-1193, Japan

Supplementary Methods

Binding affinity measurements on Biacore

The Syn–tubulin binding affinity constants were determined using surface plasmon resonance analysis (SPR) on a Biacore T200 system. For this purpose, purified tubulin was biotinylated (341-06801, Dojindo. Japan), immobilized onto the surface of an SA sensor chip (BR-1005-31; GE Healthcare Bio-Sciences, UK), and subsequently incubated with different concentrations of purified Syns in BRB80 buffer. Binding was allowed to proceed for 1 min at 30 µl/min, followed by a 5-min dissociation step at a rate of 30 µl/min and a temperature of 20°C. Each analytical cycle comprised injection of 88 µl of Syns in 2 series of increasing concentrations (2, 4, 8, 16, and 32 µM for αSyn, βSyn, and γSyn; 5, 10, 20, 40, and 80 µM for mutated αSyn) in BRB80 buffer. The assay was performed using a single-cycle kinetic approach on Biacore T200. Biacore software was used to fit the binding curves according to a 1:1 binding model.

MT pelleting assay

The co-pelleting of Syns with MTs was examined using the following co-sedimentation procedure: 4 µM of each synuclein was mixed with 2 µM MTs stabilized with paclitaxel and incubated for 30 min at room temperature. The MTs were ultracentrifuged at 100,000 × *g* for

10 min at 25°C, and the supernatants and pellets were subjected to SDS-PAGE (15% polyacrylamide) and CBB staining.

DRG preparation and culture

DRG neuron preparation from postnatal day 3-5 mice was performed as described previously [1]. Immediately after dissection, DRGs were transfected with expression vectors for various proteins using the Neon transfection system (Thermo Fisher Scientific, MA, USA) or lentivirus-mediated expression system. The cells were plated onto poly-L-lysine (Sigma, MO, USA) and matrigel (Corning, NY, USA) precoated glass bottom dishes (Iwaki Glass Inc., Tokyo, Japan) and cultured in F-12 medium (Wako Chemicals, Osaka, Japan) supplemented with 20 ng/ml 2.5S mNGF (Sigma-Aldrich, MO, USA), 1% B27 (Thermo Fisher Scientific, MA, USA) for 24-48 h.

Reconstruction of superresolution image

Single molecule localization microscopy was setup on an inverted microscope (Ti-E, Nikon, Japan) with optics to enable epi-illumination of 405 nm (Excelsior-405C-50-CDRH, Spectra-Physics, USA), 488 nm (CYAN-488-50-CDRH, Spectra-Physics), and 561 nm (ECLSR-561-

SH-W, Spectra-Physics) with dichroic mirror (Di01-R405/488/561/635, Semrock, USA). Fluorescence signals were detected by 100x objective lens (numerical aperture 1.49, ApoTIRF, Nikon, Japan). To obtain CAGE500 and CAGE590 fluorescence signals simultaneously, dual view optics (W-VIEW GEMINI, Hamamatsu Photonics K.K., Japan) with dichroic mirror (FF605-Di01-25x36, Semrock, USA) and emission filters (FF01-520/35-25 and FF01-628/40-25 for CAGE500 and CAGE590, respectively, Semrock, USA) were equipped to our system. Images were recorded by scientific Complementary MOS camera (ORCA-Flash4.0V2, Hamamatsu photonics K.K., Japan) and image acquisition software (HCImageLive, Hamamatsu Photonics K.K., Japan). To activate CAGE-dyes periodically, 405 nm was illuminated periodically by using mechanical shutter (SH05, Thorlabs Inc., USA) which is controlled by LabVIEW software and data acquisition module (LabVIEW2012 and USB-6211, National Instruments, USA).

5000 frames of single molecule images were processed by ImageJ with ThunderSTORM plugin [2]. After reconstruction, both CAGE500 and CAGE590 derived superresolution images were aligned by using StackReg plugin [3] of ImageJ with Affine transformation.

Cryo-electron microscopy

Purified tubulin and Syns were mixed at a molar ratio of 1:1, diluted with BRB80 buffer to a tubulin concentration at 2.5 μM, and incubated with 1 mM GTP at 37°C for 30 min. To avoid the influence of paclitaxel on the pfs number [4], the MTs were not stabilized with paclieaxel. The polymerized MTs were loaded onto a hydrophilized holy molybdenum grid (R3.5/1, Mo #200, Quantifoil, Germany) and embedded into an amorphous thin ice layer by quick freezing. Electron micrographs were recorded using a TEM (EF2000, Hitachi, Japan) equipped with a 2k × 2k CCD camera (TVIPS, Germany) with under-focus values of 2.5-5 μm. The magnification of the micrographs was 130,000×, with a sampling interval of 1.94 Å and an electron dose of approximately 14 e/Å^2. Image processing was performed using the Extensible object-oriented system (Eos) [5] and ImageJ. All TEM images were subjected to contrast transfer function (CTF) corrections, through which the images were compensated using phase flipping with consideration of the CTFs following the estimated defocusing values using "ctfDisplay".

Supplementary Figure Legends

Supplementary Figure S1. Summary of the LC-MS/MS analysis. (**a**) Interactorme of βIII-tubulin assigned by the LC-MS/MS analysis. Candidate interactors are listed in order of higher "unused score" from the data list of the βIII-tubulin interactome. αSyn and γSyn were selectively detected in the ligated femoral nerve. Three independent experiments were performed. (**b**) Generation of an antibody specific for murine γSyn. This antibody specifically recognizes murine γSyn as confirmed by the disappearance after absorption of the antiserum with the murine recombinant γSyn that was used for immunization. β-actin was used as a loading control. (**c**) Brain extract was incubated with or without alkaline phosphatase, and incubation with alkaline phosphatase was shown to shift the electrophoretic mobility of γSyn. The filled arrowhead indicates phosphorylated γSyn, and the open arrowhead indicates unphosphorylated γSyn.

Supplementary Figure S2. Analysis of the binding affinity of Syn to tubulin. (**a**) Prepared recombinant αSyn, βSyn, and γSyn. Each bacterially expressed GST-fusion Syn used in this experiment was treated with thrombin and confirmed by CBB staining. (**b**-**h**) Binding affinities of Syns for tubulin were examined by surface plasmon resonance on a Biacore™-T200

instrument. Sensorgram plots of the binding of recombinant Syns to tubulin are shown. One sensorgram (colored curve) is from a run in single-cycle kinetics mode. In this mode, the analyte is injected at increasing concentrations in a single cycle and the surface is not regenerated between injections. The data were fitted to a 1:1 binding model (black curves). (**i**) Biacore binding affinities of Syns to tubulin. (**j-l**) Protein–protein interactions of αSyn, βSyn, and γSyn. The sensorgram plots for each Syn indicated a lack of obvious interactions between the Syns under our conditions. (**m,n**) MT pelleting assays with Syns. Syns were incubated with or without MTs and subjected to CBB staining. Consistent with the results of a previous study [6], αSyn interacted with MTs. In addition, βSyn, γSyn and αSyn S129A exhibited binding affinity for MTs, although αSyn S129E, A30P and E46K did not. S: Supernatant; P: Precipitate.

Supplementary Figure S3. Expression of Syns, tau and DCX in DRG. Exogenously expressed mChe-αSyn (**a**,**b**), mChe-γSyn (**d**,**e**), mChe-tau (**g**,**h**) and mChe-DCX (**j**,**k**) were confirmed by WB (a,d,g,j), and shown to be present within the axons of DRG neurons (b,e,h,k). The endogenous expression of each target was also confirmed by WB (open arrowheads in a, d, g and j), or by immunostaining with antibodies against αSyn (**c**), γSyn (**f**), tau (**i**) and DCX (**l**). The areas surrounded by yellow rectangles are shown at higher magnification in the lower

panel. The yellow arrows indicate the punctate distributions of each target within the axon. Scale bars: 5 μm in (b), (e), (h) and (k); and 10 μm in (c), (f), (i) and (l).

Supplementary Figure S4. SR-SIM images of axons. Co-localization of mNG-DIC1, mChe-αSyn and mTQ-βIII-tubulin (**a**), mNG-LIS1, mChe-αSyn and mTQ-βIIItubulin (**b**), and mNG-mNudC, mChe-αSyn and mTQ-βIII-tubulin (**c**) were observed by SR-SIM. The white arrowheads indicate co-localized particles. S and P indicate the direction of the neuronal soma and the periphery of DRG neurons, respectively. Scale bar, 10 μm.

Supplementary Figure S5. SR-PALM images of axons. (**a-c**) Averaged and SR-PALM images of axons were visualized using antibodies against βIII-tubulin and DIC1 (a), βIII-tubulin and αSyn (b), or DIC1 and αSyn (c). The averaged images simulate those obtained by conventional fluorescence microscopy, in which tubulin fluorescence was blurred and appeared as indistinct blobs. In contrast, SR-PALM images sharply visualized short MT fragments along axons. The white square shows the regions of interest that are enlarged in Fig. 3g-i. (**d**) Enlarged SR-PALM image of (a). The yellow arrows indicate free dynein particles without βIII-tubulin fragments. Scale bars: 5μm in (a-c); and 1 μm in (d).

Supplementary Figure S6. Effect of detergent on the tubulin pull-down assay. Anti-βIII-tubulin antibody pre-bound to Protein G-Sepharose was incubated with cell extracts from DRG neurons with or without 0.1% Triton X-100. After centrifugation, the supernatants and precipitates were collected and subjected to WB analysis. The graph shown was obtained from three independent experiments.

Supplementary Figure S7. Evaluation of siRNA-mediated depletion of αSyn and γSyn. (**a**) siRNA against αSyn (left) or γSyn (right) efficiently depleted endogenous synucleins, as confirmed by WB. β-actin was used as a loading control. The quantification data for each siRNA from 3 independent sets of experiments are shown in the lower panel. The signal intensity of αSyn or γSyn in the control siRNA treated sample was set at 100%. The percentages of each sample are given as signal intensity ratios. The data are presented as the mean ± SEM; *p* values were calculated using analysis of variance, ***$p < 0.001$, "NS" means not significant. (**b**) Effect of αSyn and γSyn in dynein distribution. After siRNAs treatment, endogenous dynein distribution in the DRG axon was probed using an anti-DIC1 antibody. Each phase-contrast image is shown in the lower panel. Scale bar: 10 μm.

Supplementary Figure S8. Effect of Synus on tubulin polymerization examined by dark-field light microscopy. Galleries of polymerized MTs obtained under the conditions indicated in the upper side of each image set.

Supplementary Figure S9. Effect of Syns on tubulin polymerization examined by TEM. (**a**) MTs polymerized at 40 μM tubulin. (**b**) MTs captured at higher magnification. (**c**-**e**) Tubulin concentration at 5 μM failed to polymerize MTs (c). Addition of 0.4 mg/ml BSA (d) or GST (e) to 5 μM tubulin did not facilitate tubulin polymerization. (**f**) Higher magnification of (a). (**g**-**m**) MT polymerization was performed at 5 μM tubulin with αSyn (g), βSyn (h), γSyn (i), αSyn S129A (j), αSyn S129E (k), αSyn A30P (l), or αSyn E46K (m). (**n)** Numbers of surface MTs observed in a 10.9 μm × 10.9 μm field were measured (N = 20 in each condition). The data are presented as the mean ± SEM; *p* values were calculated by *t*-test, ****p* < 0.001, "NS" means not significant. Scale bar: 2 μm in (a), (c), (d) and (e); and 100nm in (b) and (f-m).

Supplementary Figure S10. Colloidal gold particle-based characterization of synuclein binding to MTs. (**a**) To verify specific labeling, colloidal gold was mixed with MT solution in

the absence of His-αSyns. Here, no specific binding of colloidal gold particles to MTs was detected. (**b**,**c**) Galleries of His-αSyn binding to MTs with different concentrations. (**d**,**e**) Galleries of His-αSyn binding to MTs with string-like (d) and necklace-like (e) binding confirmed with tilting TEM images as described in Supplementary Fig. S11. (**f**,**g**) Galleries of His-βSyn (f) and His-γSyn (g) binding to MTs. Scale bars: 100 nm in (a); and 30 nm in (b-g).

Supplementary Figure S11. Stereo images showing the topography of αSyn bound to MTs. (**a**) Schematic drawing showing a comparison of the stereo-pairs. When the gold particles are located on the same flattened surface of a MT, tilting produces a compressed image of the width vertical to the tilting axis (upper). If the particles are located on different surfaces of a MT, tilting causes deformation of the visual alignment (lower). (**b**,**c**) Stereo pairs of Gold-αSyn bound MTs. The electron beam is approximately normal to the carbon surface (left) and the surface has been tilted (middle) by 60°. The simulated images are shown with their compressed width at cos 60° vertical to the tilting axis (right). Simulated tilting images were overlapped with the images taken after actual tilting. When the visual alignment of gold particles in the tilted image (middle) and the simulated image (right) were similar, the particles were considered to be located on the flattened carbon surface (b). When the tilted image (middle) differed from

that in the simulated image (right), the gold particles were considered to be located at the different surface, on which Gold-αSyns were encircling the MT (c) [7].

Supplementary Figure S12. TEM analyses of the synuclein bound to MTs. (**a**) Additional TEM image of MT polymerized with Halo-αSyn. Bamboo joint-like structures on the MTs are indicated by magenta arrowheads. (**b**) Additional image of the MT end structure with Halo-αSyn (blue arrowheads). (**c**) Negatively stained Gold-mNudC labeled by an anti-mNudC antibody conjugated colloidal gold. (**d**) Negatively stained image of MT with colloidal gold particles (ϕ = 10 nm). (**e**) Additional TEM image of MT bound Halo-αSyn (magenta) and Gold-mNudC (green). (**f**) Additional cryo-TEM image of MTs polymerized with Halo-αSyn. Joint-like structures on MTs are indicated by the magenta arrowheads. (**g**) Additional cryo-TEM image of MTs polymerized at 5 μM of tubulin in the presence of αSyn. The MT pfs numbers in (f) and (g) determined from Moiré patterns are indicated at the top left. Scale bar: 30 nm.

Supplementary Figure S13. Characterization of αSyn binding to MTs *in vitro*. (**a**) Two additional image sets related to Fig. 6l. (**b**) Steady-state MT binding of αSyn. Co-sedimentation assays were performed at a constant concentration of αSyn and various concentrations of MT

(filled circles). The smooth curve displays the best fits of the data to a hyperbola and indicates the dissociation constants (K_d) and maximum bindings (B_{max}). The error bars indicate the SEM (N = 3).

Supplementary Figure S14. Galleries of unconventional MTs carrying 14-pfs in femoral nerves. Galleries of MTs with 14-pfs in unligated (**a**) and ligated (**b**) femoral nerves are shown.

Supplementary Figure S15. Original gel/blotting images showed in the manuscript. Original images of Fig. 1c (**a**), Fig. 1d (**b**), Fig. 1e (**c**), Fig. 6f (**d**), Supplementary Fig. S1b (**e**), Supplementary Fig. S1c (**f**), Supplementary Fig. S2a (**g**), Supplementary Fig. S2m (**h**), Supplementary Fig. S2n (**i**), Supplementary Fig. S3a (**j**), Supplementary Fig. S3d (**k**), Supplementary Fig. S3g (**l**), Supplementary Fig. S3j (**m**), Supplementary Fig. S6 (**n**) and Supplementary Fig. S7a (**o**) were shown. Magenta rectangle surrounding area in each image indicates cropped region.

Supplementary Video Legends

Supplementary Video 1. Live-cell imaging of mChe-tagged Syns in DRG neurons. Moving particles corresponding to mChe-αSyn, mChe-βSyn and mChe-γSyn were observed in DRG axons. Speed: 5×. Scale bar: 5 μm.

Supplementary Video 2. Live-cell imaging of mChe-tagged mutated Syns in DRG neurons. mChe-αSyn S129A particles exhibited active moving similar to that of WT αSyn. However, significantly decreased movement was observed in mChe-αSyn S129E, mChe-αSyn A30P or mChe-αSyn E46K were observed in DRG axons. Speed: 5×. Scale bar: 5 μm.

Supplementary Video 3. Live-cell imaging of mChe-tagged tau and DCX in DRG neurons. mChe-tau showed actively moving particles with the cytoskeletal background in a DRG axon, whereas mChe-DCX displayed only the fibrous structures. Speed: 5×. Scale bar: 5 μm.

Supplementary Video 4. Triple-color live-cell imaging of fluorescently tagged proteins in a DRG neuron. Co-migration of mNG-DIC1, mChe-αSyn, and mTQ-βIII-tubulin; mNG-LIS1, mChe-αSyn, and mTQ-βIII-tubulin; and mNG-mNudC, mChe-αSyn, and mTQ-βIII-tubulin in DRG axons was shown. Speed: 5×. Scale bar: 5 μm.

Supplementary Video 5. Live-cell imaging of mChe-tagged or LysoTracker-labeled proteins in DRG neurons. Robust axonal transport of mChe-DIC1, VP26-mChe and LysoTracker was inhibited by the treatment of the cells with siRNAs against αSyn and γSyn. Speed: 5×. Scale bar: 5 μm.

Supplementary Video 6. Dark-field imaging of the pivot movement of MTs on a coverslip. Spontaneous depolymerization of MTs was observed after MTs were captured on a coverslip. The yellow arrow indicates a nodal point of a pivoted MT. Speed: 5×. Scale bar: 5 μm.

Supplementary Video 7. Dark-field imaging of the depolymerizing MTs on a coverslip. Spontaneous depolymerization of MTs was observed in the absence of αSyn and in the presence of αSyn or αSyn E46K. The yellow arrows indicate the depolymerizing ends of the MTs at the start of the movie. Speed: 5×. Scale bar: 5 μm.

References

1 Winter, J., Forbes, C. A., Sternberg, J. & Lindsay, R. M. Nerve growth factor (NGF) regulates adult rat cultured dorsal root ganglion neuron responses to the excitotoxin capsaicin. *Neuron* **1**, 973-981 (1988).

2 Ovesny, M., Krizek, P., Borkovec, J., Svindrych, Z. & Hagen, G. M. ThunderSTORM: a comprehensive ImageJ plug-in for PALM and STORM data analysis and super-resolution imaging. *Bioinformatics* **30**, 2389-2390, doi:10.1093/bioinformatics/btu202 (2014).

3 Thevenaz, P., Ruttimann, U. E. & Unser, M. A pyramid approach to subpixel registration based on intensity. *IEEE transactions on image processing : a publication of the IEEE Signal Processing Society* **7**, 27-41, doi:10.1109/83.650848 (1998).

4 Andreu, J. M. *et al.* Low resolution structure of microtubules in solution. Synchrotron X-ray scattering and electron microscopy of taxol-induced microtubules assembled from purified tubulin in comparison with glycerol and MAP-induced microtubules. *Journal of molecular biology* **226**, 169-184 (1992).

5. Yasunaga, T. & Wakabayashi, T. Extensible and object-oriented system Eos supplies a new environment for image analysis of electron micrographs of macromolecules. *J Struct. Biol.* **116**, 155-160 (1996).

6. Cartelli, D. *et al.* alpha-Synuclein is a Novel Microtubule Dynamase. *Scientific reports* **6**, 33289, doi:10.1038/srep33289 (2016).

7. Olins, A. L., Wilkinson-Singley, E., Bentley, J. & Olins, D. E. Stereo-electron microscopy and energy-dispersive x-ray analysis of avian reticulocytes. *European journal of cell biology* **19**, 239-249 (1979).

Toba and Jin et al., Supplementary Figure S1

a Identified proteins by LC-MS/MS

Protein no.[a]	Protein identification[b]	Accession no.[c]	% coverage[d]	Unused[e]
1	Tubulin beta-3 chain	gi | 20455323	84.0	46.26
2	Tubulin alpha-3 chain	gi | 135418	94.7	30.45
6	Tubulin beta-2B chain	gi | 74761283	85.2	18.01
22	Tubulin alpha-1C chain	gi | 55976169	87.5	5.27
25	Tubulin beta-4B chain	gi | 81892373	86.5	4.96
30	Gamma-synuclein	gi | 122066261	91.9	3.32
42	Alpha-synuclein	gi | 586065	66.4	2.08

[a]Order of higher values from data list. [b]Identified proteins are listed. [c]GI accession number in NCBI Protein database. [d]Percent sequence coverage based on the presence of peptides. [e]ProteinPilot unused score reflects the amount of total, unique peptide evidence related to a given protein. This table contains the identified proteins of the bIII-tubulin interactome with the score above 2.0, which is equivalent to a protein confidence level greater than 99%.

b

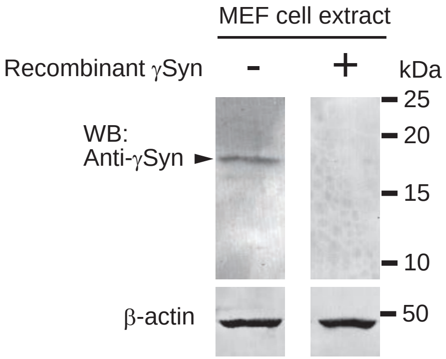

c

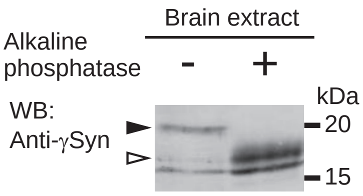

Toba and Jin et al., Supplementary Figure S2

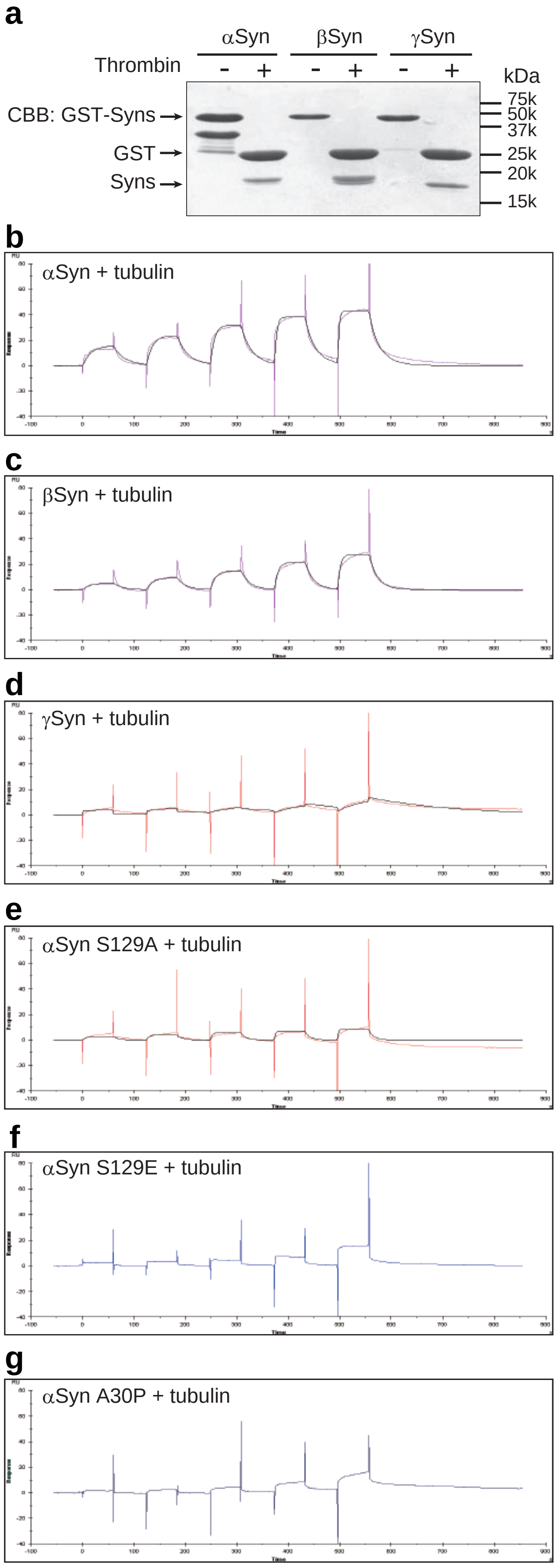

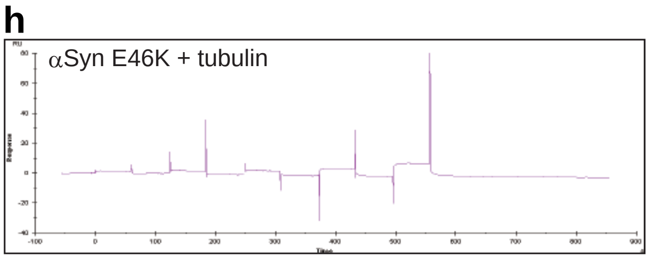

i

Protein	k_{on} (1/Ms)	k_{off} (1/s)	K_d (M)
α-synuclein	1.55×10^{4}	6.06×10^{-2}	3.90×10^{-6}
α-synuclein S129A	8.74×10^{2}	1.07×10^{-1}	1.22×10^{-5}
α-synuclein S129E*	-	-	$> 10^{-4}$
α-synuclein A30P*	-	-	$> 10^{-4}$
α-synuclein E46K*	-	-	$> 10^{-4}$
β-synuclein	5.19×10^{3}	6.02×10^{-2}	1.16×10^{-5}
γ-synuclein	2.00×10^{2}	5.96×10^{-3}	2.97×10^{-5}

*Too weak to measure.

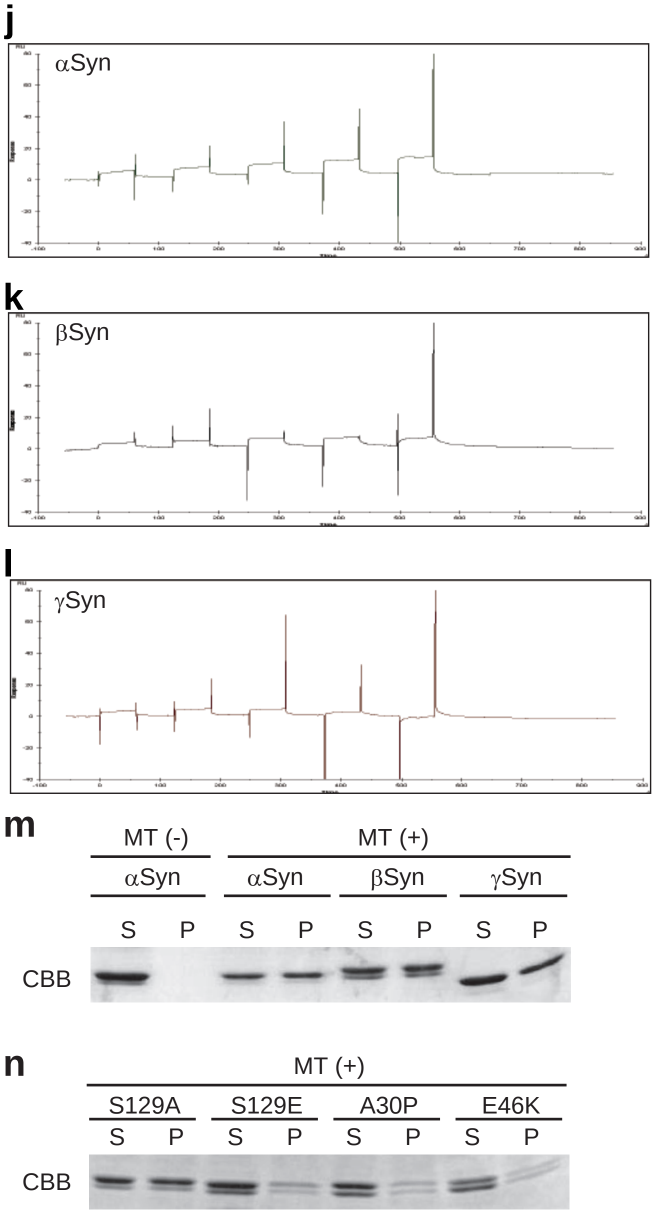

Toba and Jin et al., Supplementary Figure S3

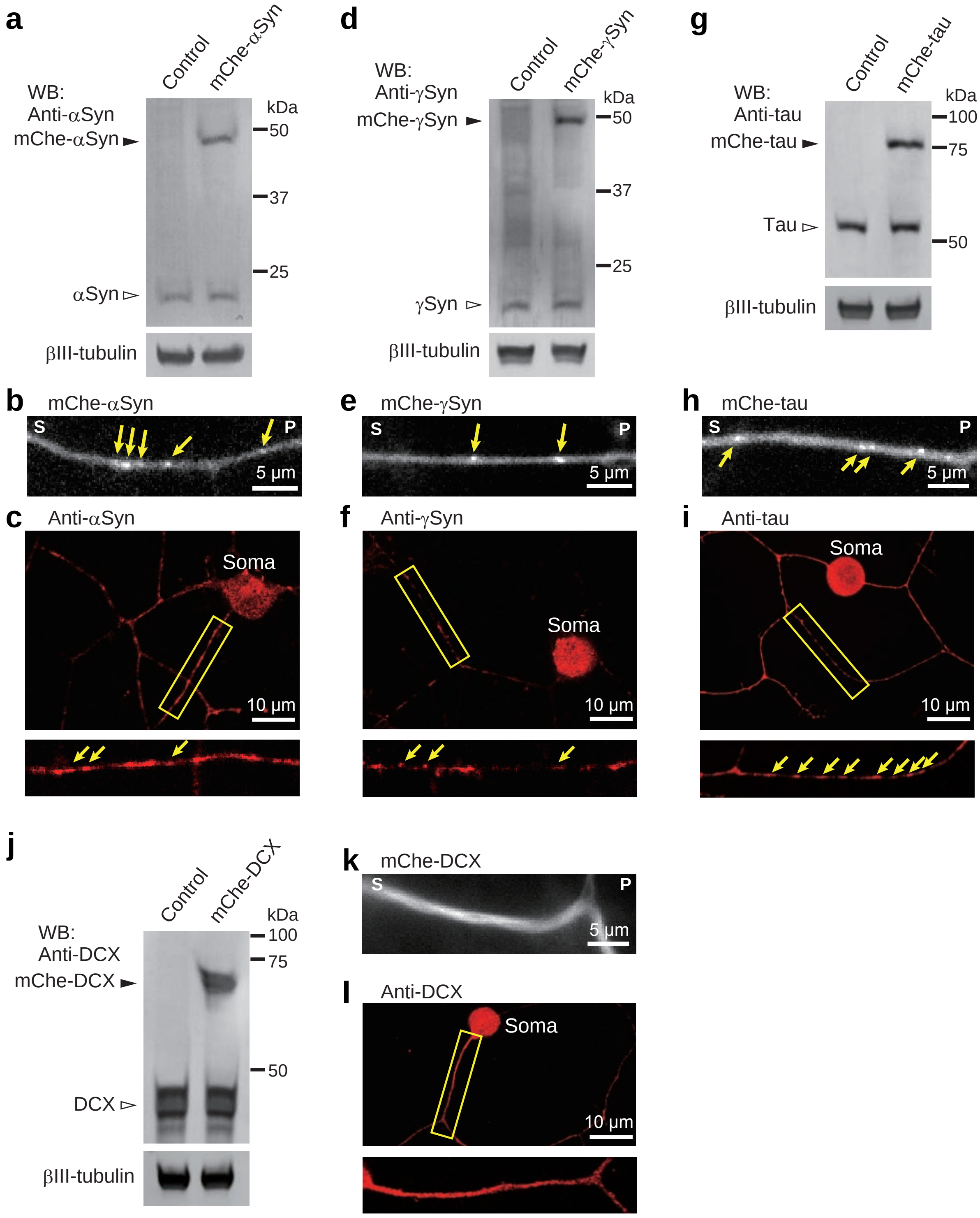

Toba and Jin et al., Supplementary Figure S4

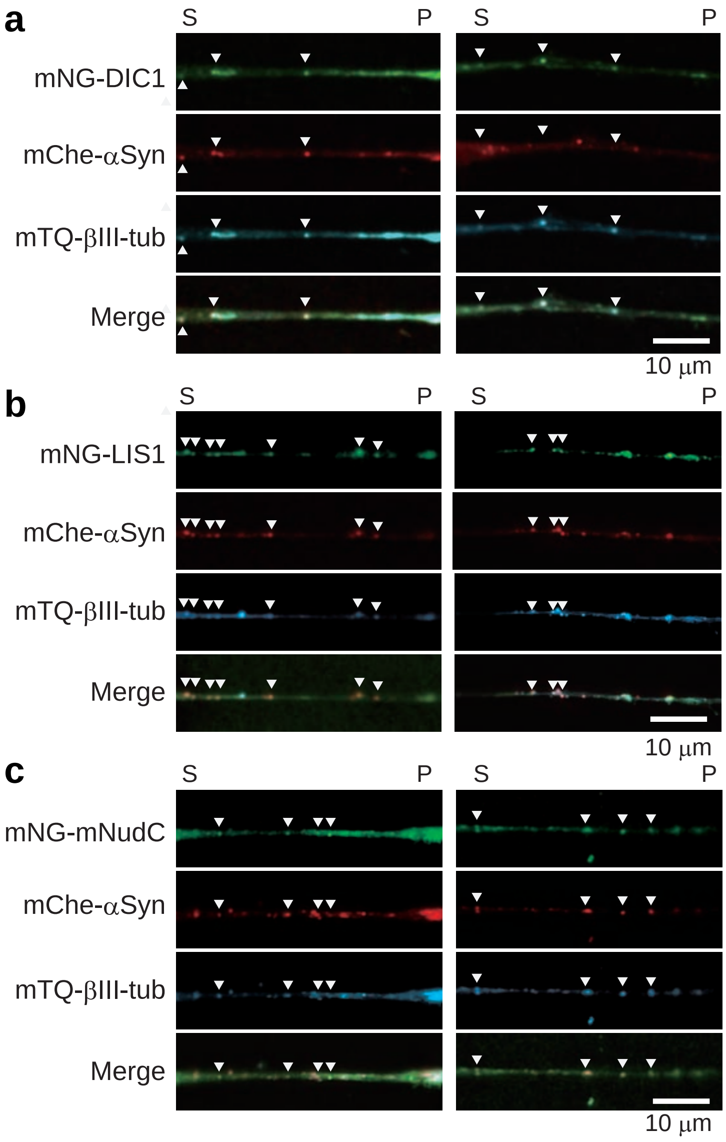

Toba and Jin et al., Supplementary Figure S5

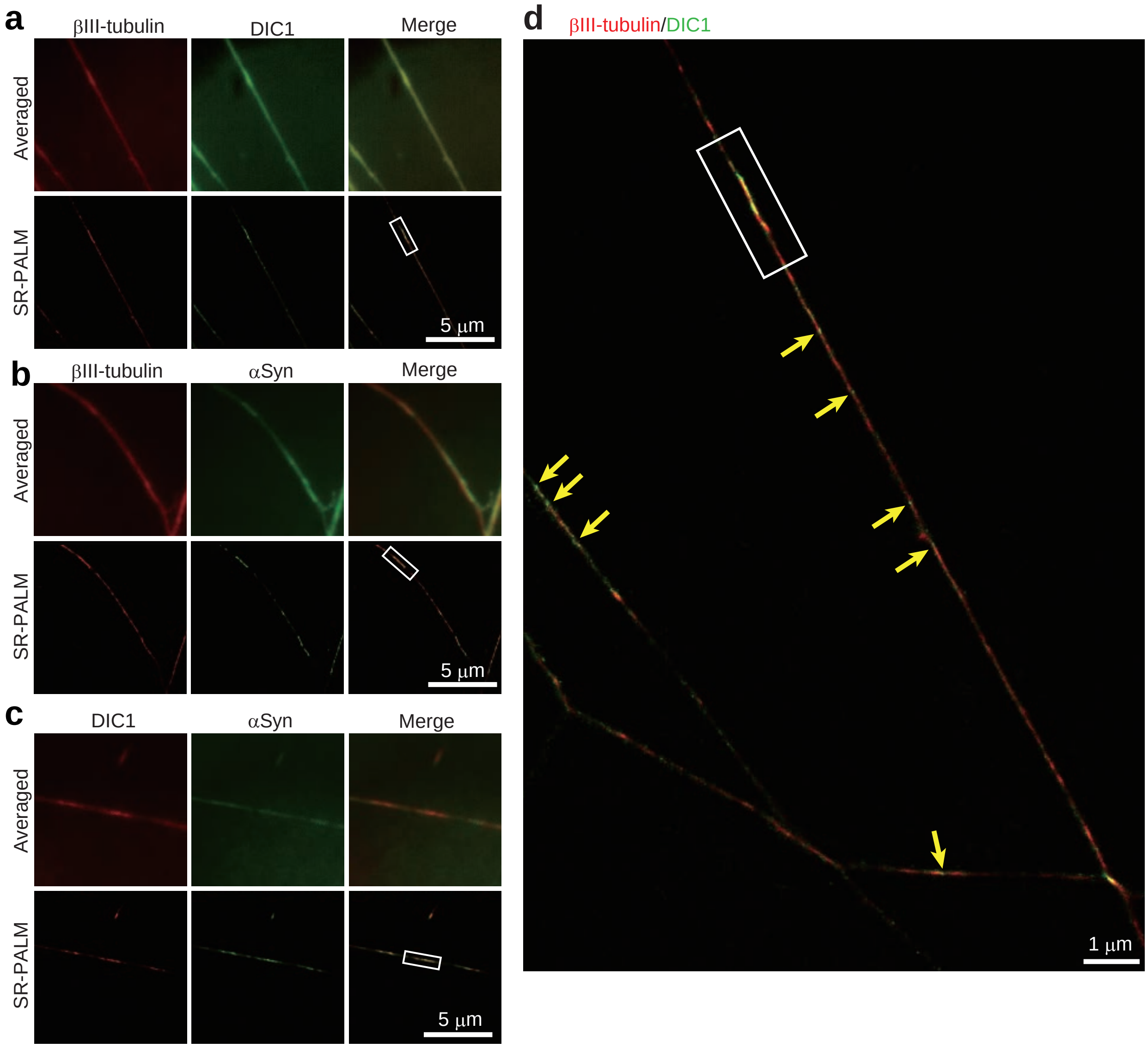

Toba and Jin et al., Supplementary Figure S6

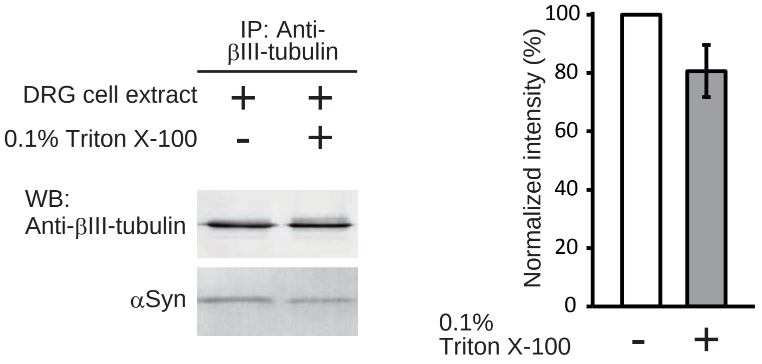

Toba and Jin et al., Supplementary Figure S7

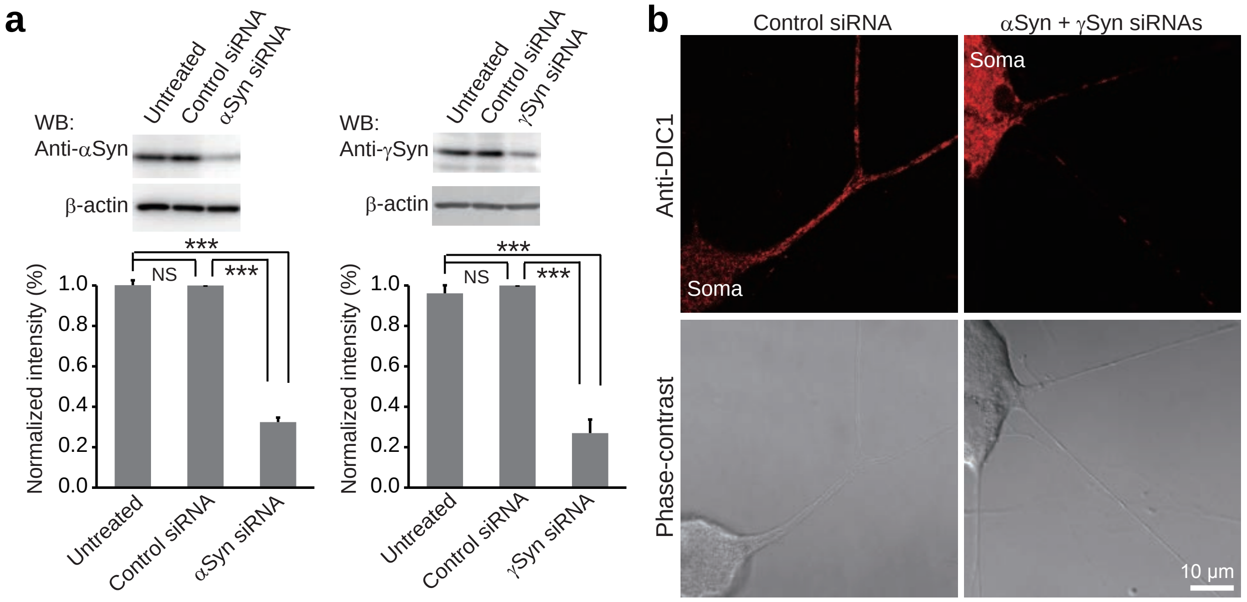

Toba and Jin et al., Supplementary Figure S8

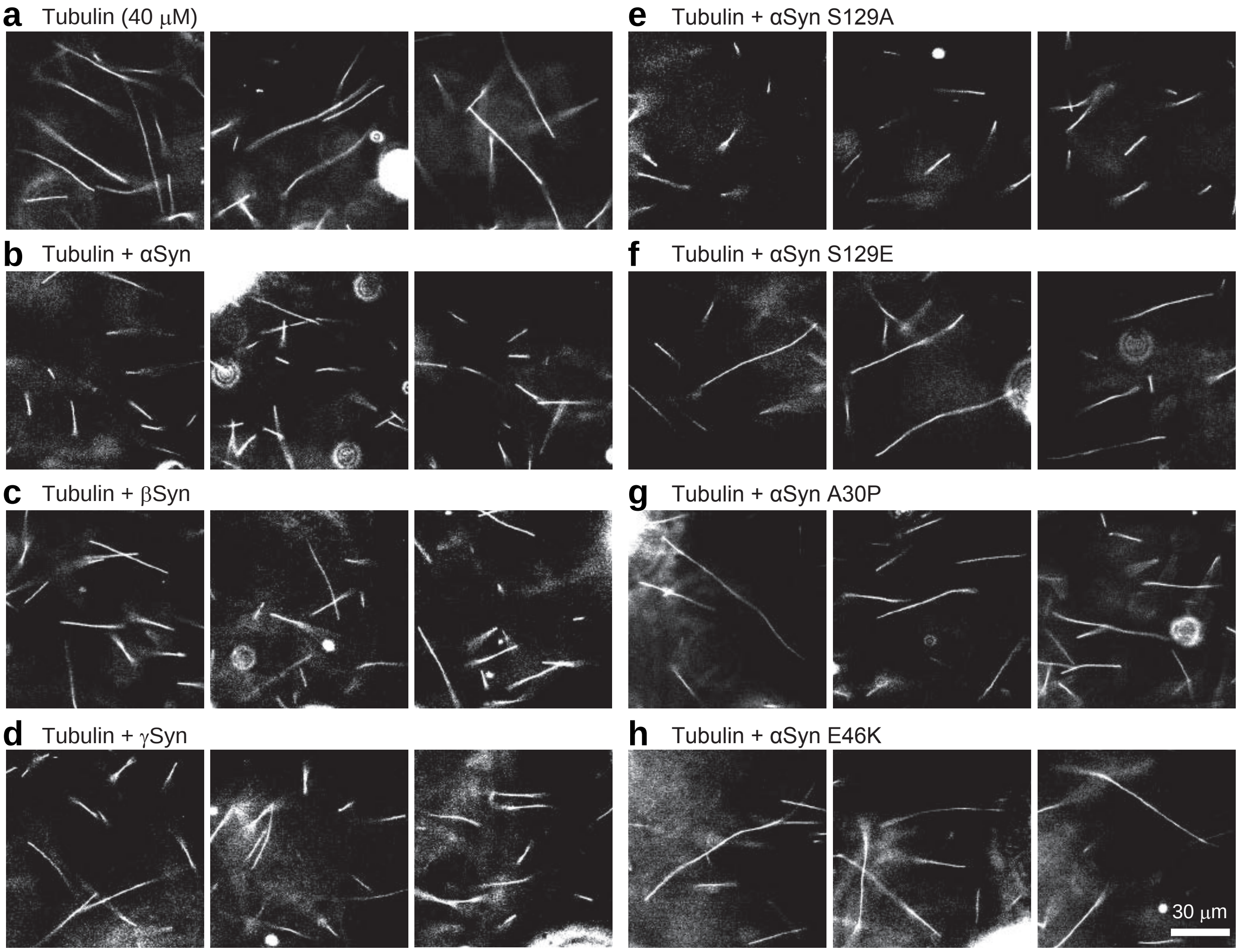

Toba and Jin et al., Supplementary Figure S9

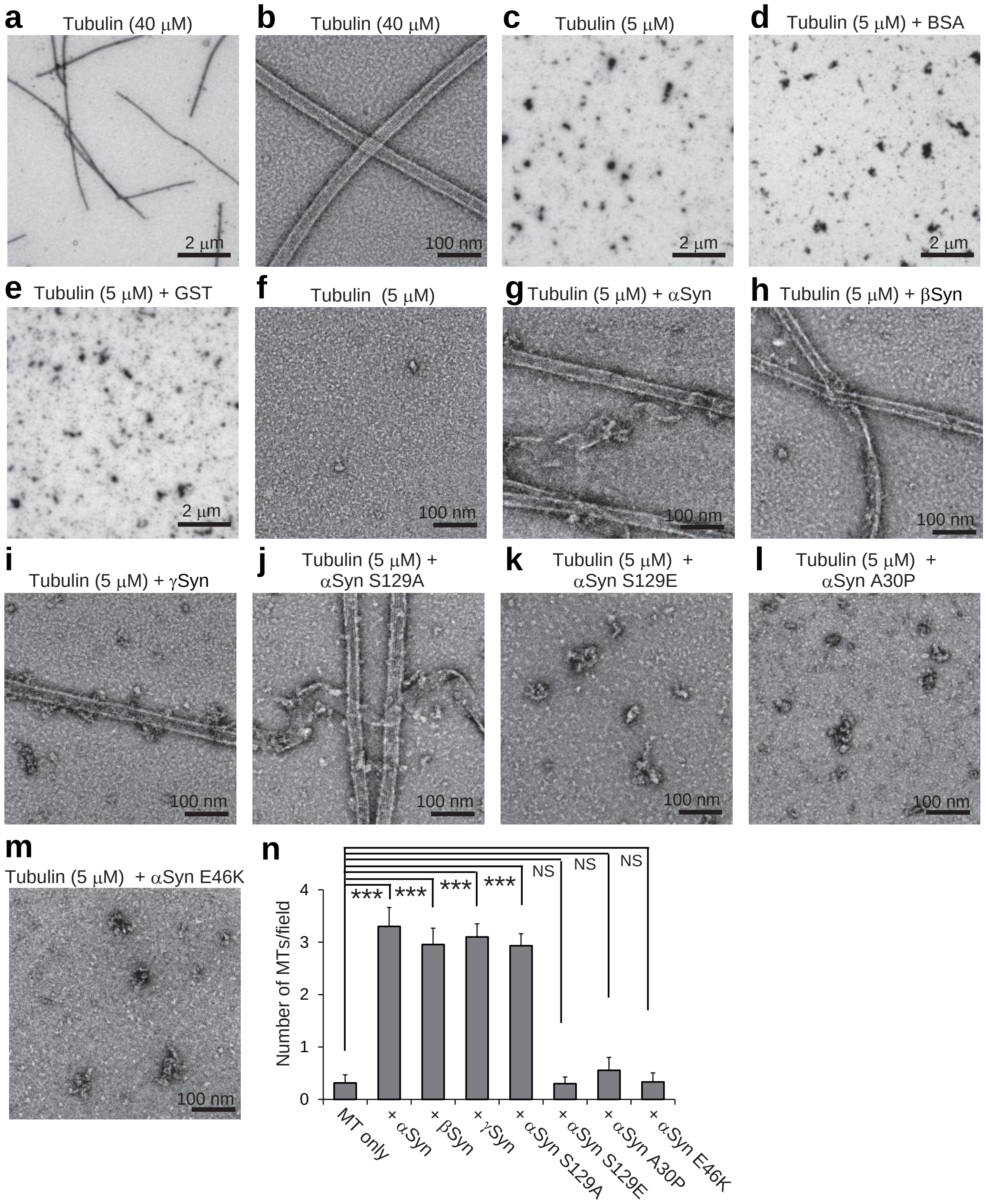

Toba and Jin et al., Supplementary Figure S10

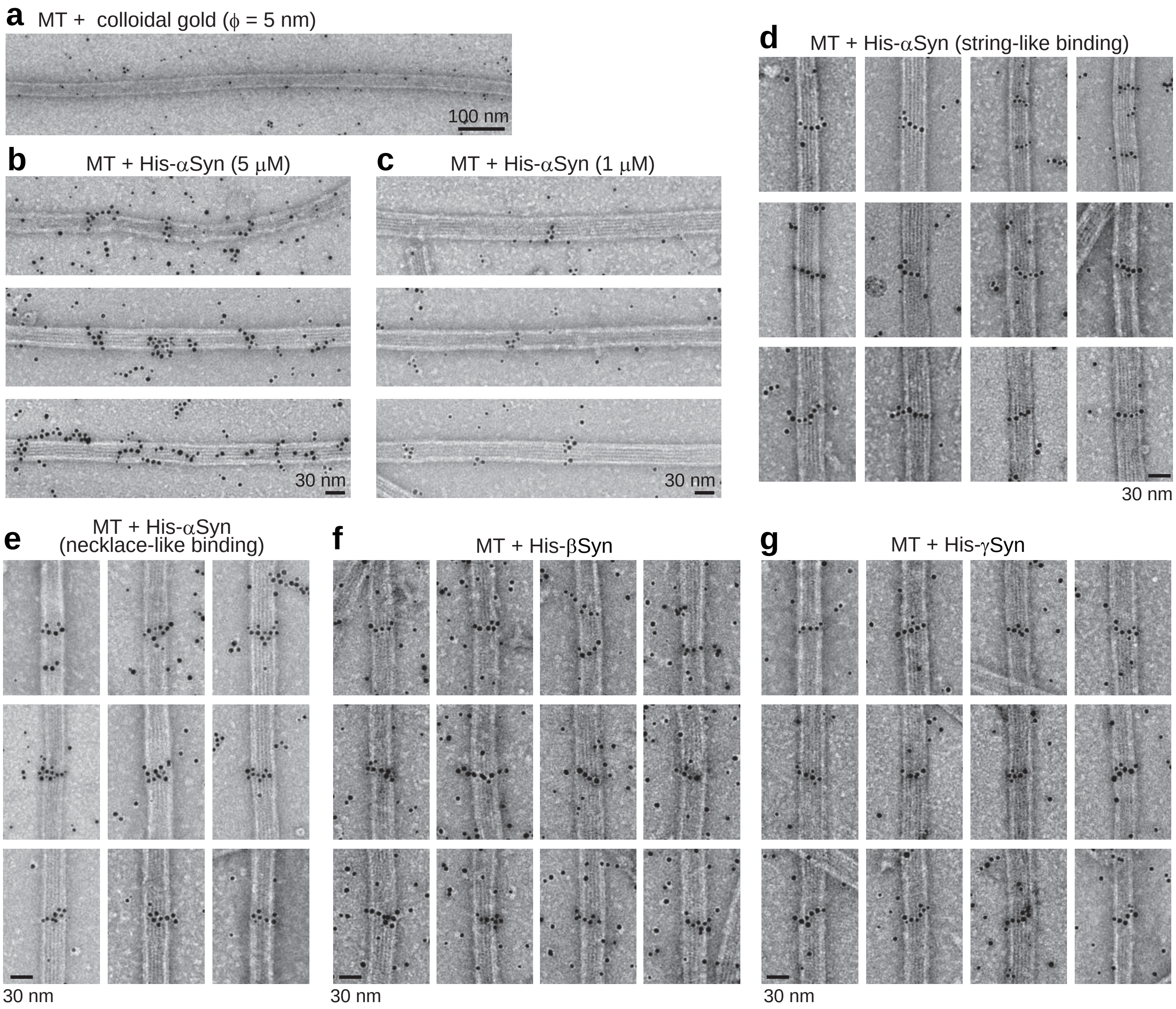

Toba and Jin et al., Supplementary Figure S11

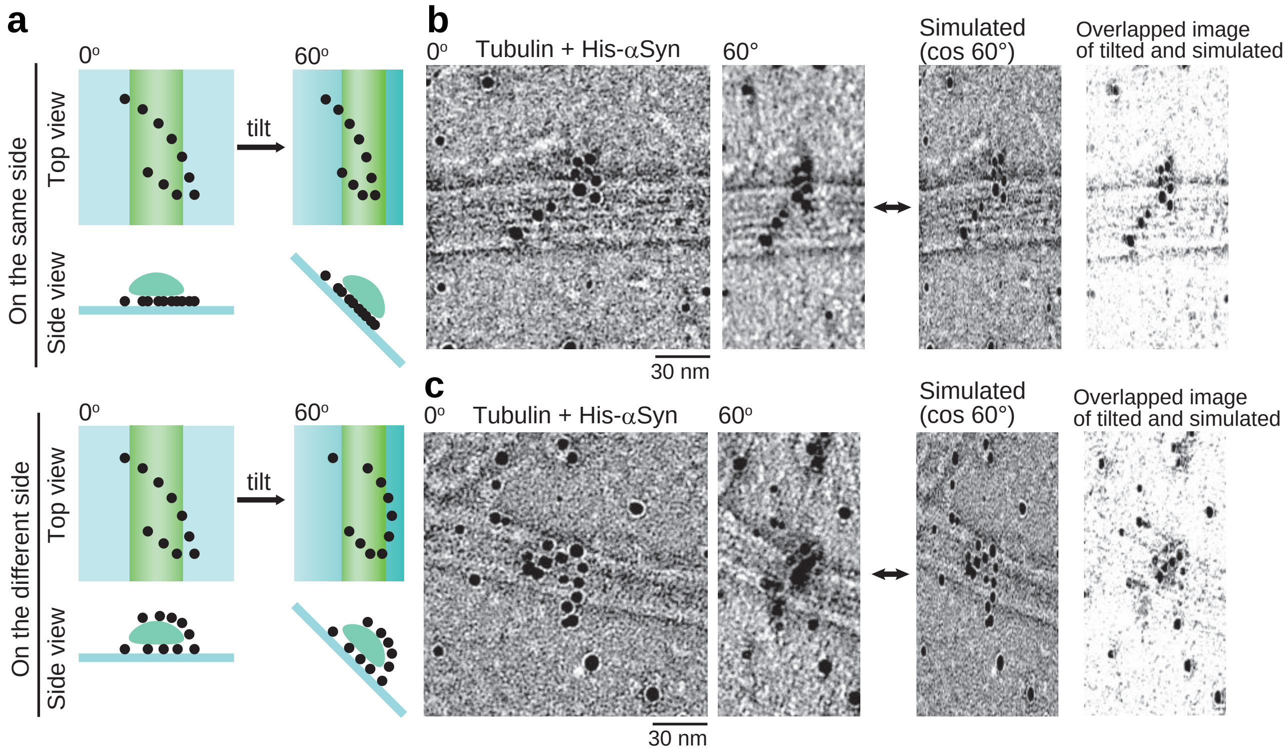

Toba and Jin et al., Supplementary Figure S12

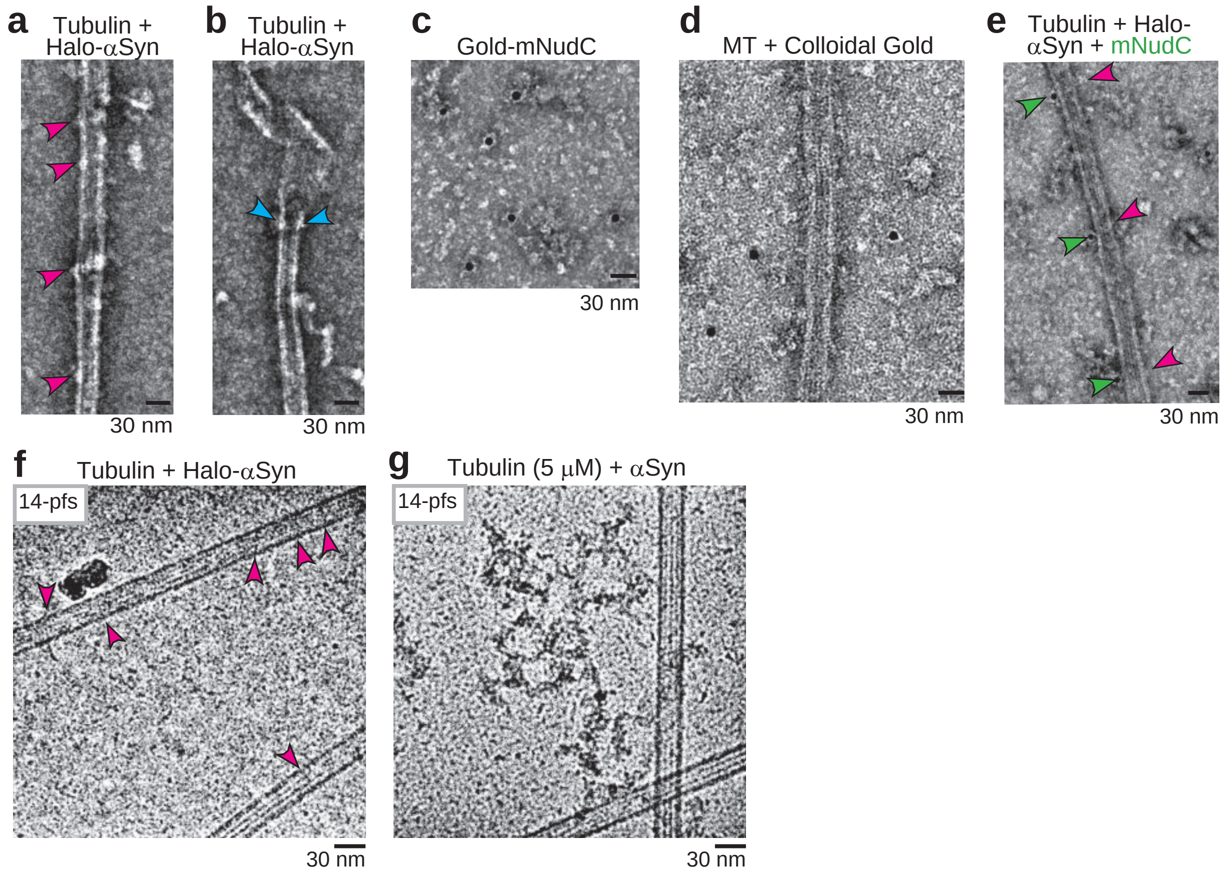

Toba and Jin et al., Supplementary Figure S13

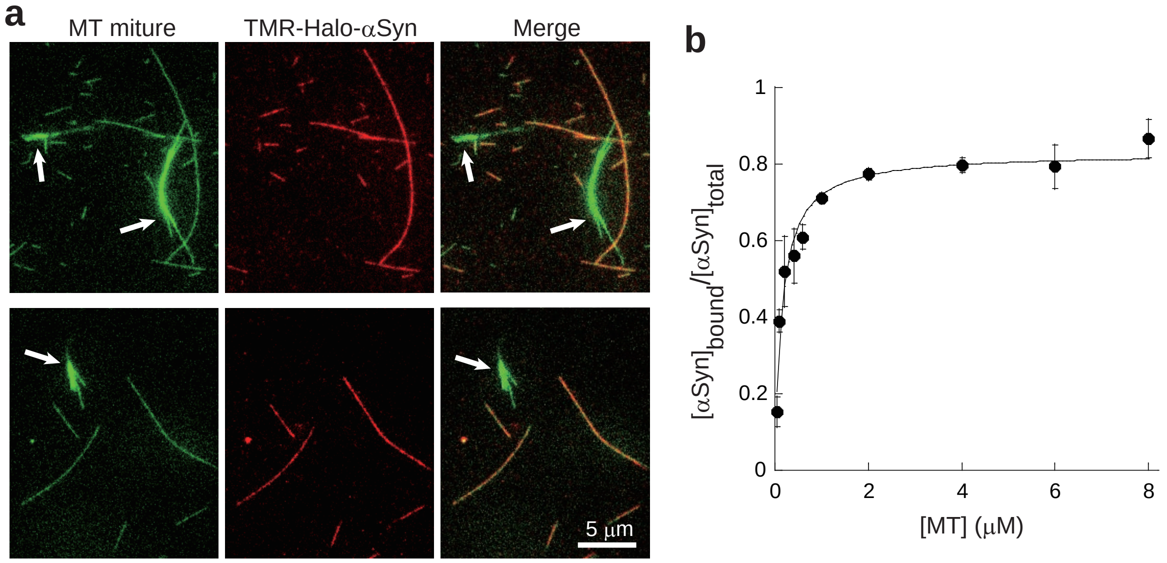

Toba and Jin et al., Supplementary Figure S14

a

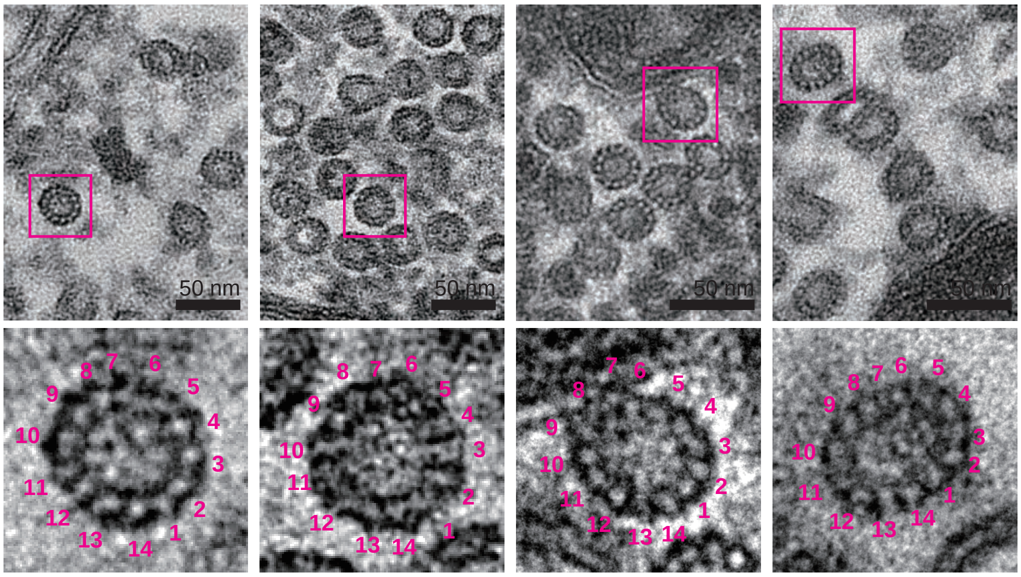

b

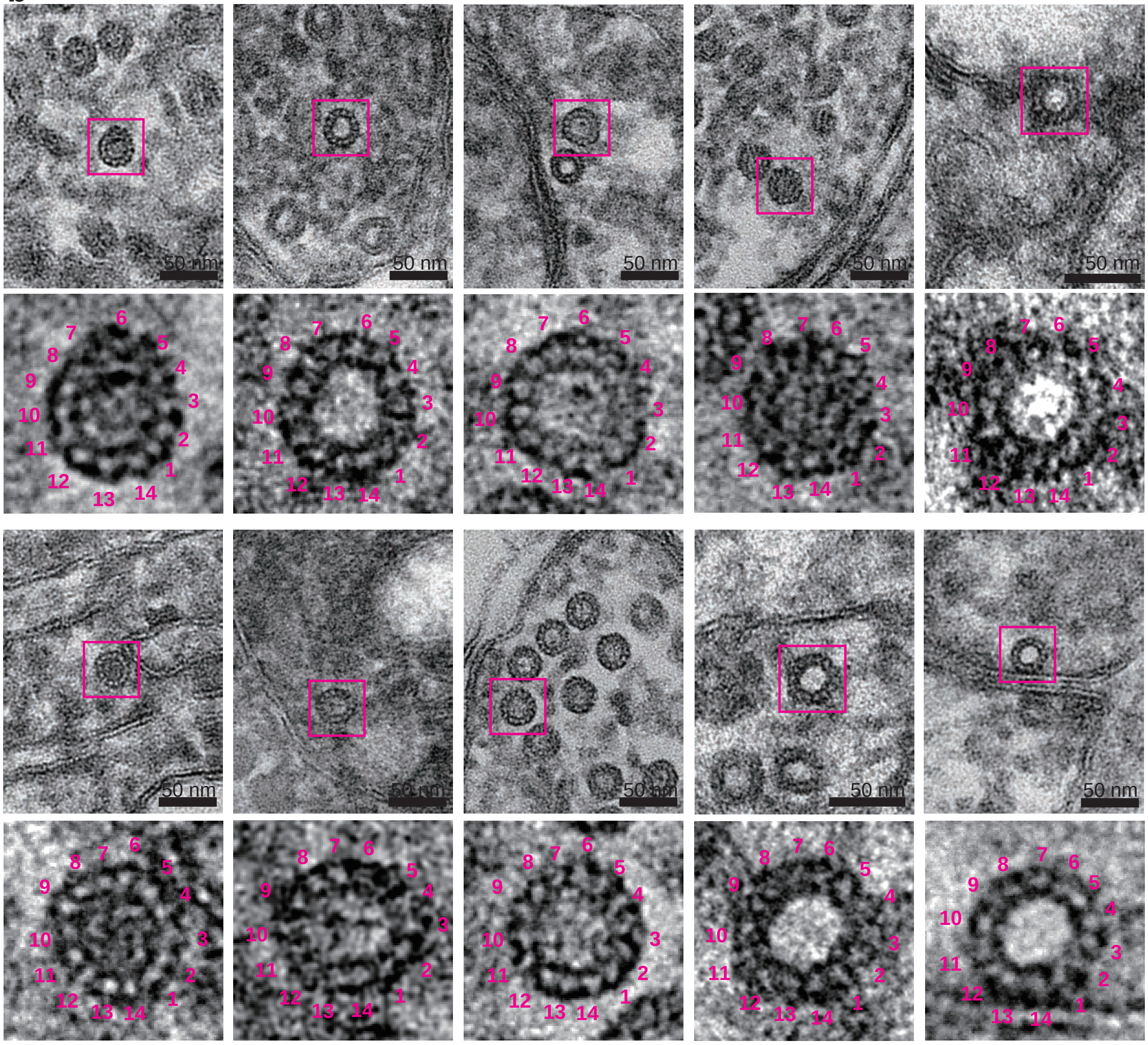

Toba and Jin et al., Supplementary Figure S15a-i

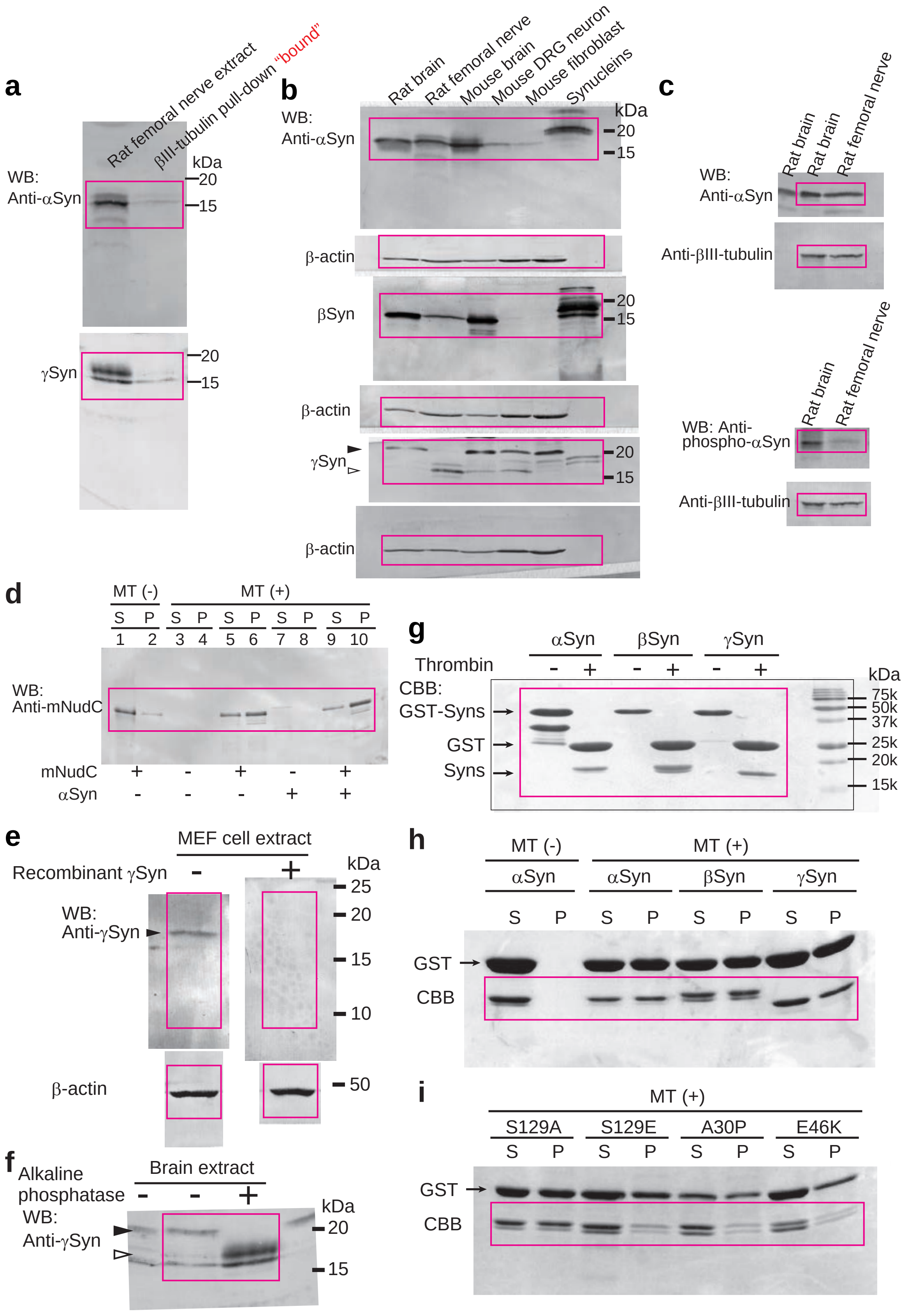

Toba and Jin et al., Supplementary Figure S15j-o

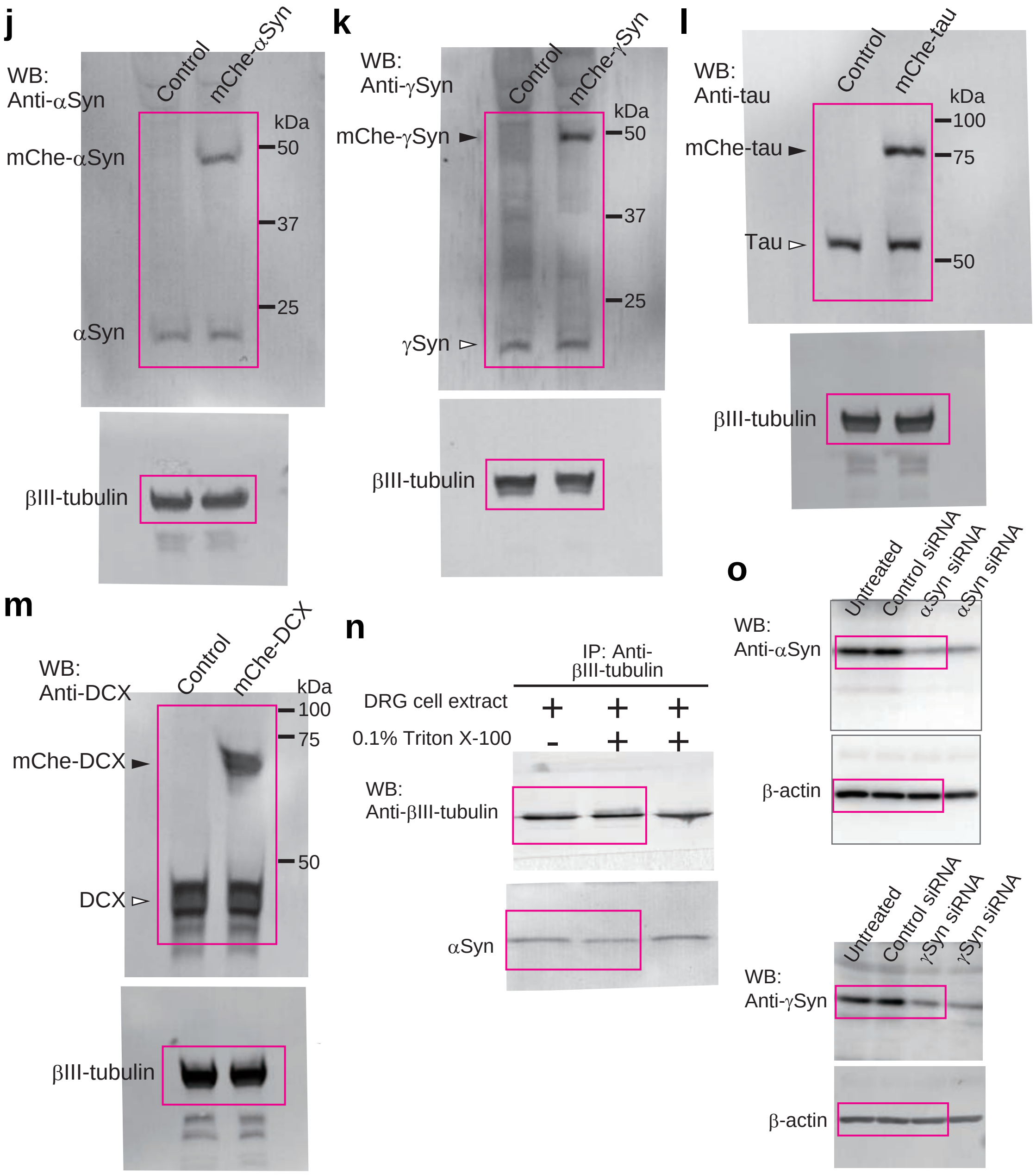

Supplement: Supplementary file 1 — Supplementary figures [file 41598_2017_15575_MOESM1_ESM.pdf]
